# Distinct functions of the laminin β LN domain and collagen IV during cardiac extracellular matrix formation and stabilization of alary muscle attachments revealed by EMS mutagenesis in *Drosophila*

**DOI:** 10.1186/1471-213X-14-26

**Published:** 2014-06-17

**Authors:** Dominik Hollfelder, Manfred Frasch, Ingolf Reim

**Affiliations:** 1 Department of Biology, Division of Developmental Biology, Friedrich-Alexander University of Erlangen-Nürnberg, Staudtstr. 5, 91058, Erlangen, Germany

**Keywords:** Extracellular matrix, Heart tube, Alary muscles, Laminin, Collagen

## Abstract

**Background:**

The *Drosophila* heart (dorsal vessel) is a relatively simple tubular organ that serves as a model for several aspects of cardiogenesis. Cardiac morphogenesis, proper heart function and stability require structural components whose identity and ways of assembly are only partially understood. Structural components are also needed to connect the myocardial tube with neighboring cells such as pericardial cells and specialized muscle fibers, the so-called alary muscles.

**Results:**

Using an EMS mutagenesis screen for cardiac and muscular abnormalities in *Drosophila* embryos we obtained multiple mutants for two genetically interacting complementation groups that showed similar alary muscle and pericardial cell detachment phenotypes. The molecular lesions underlying these defects were identified as domain-specific point mutations in *LamininB1* and *Cg25C*, encoding the extracellular matrix (ECM) components laminin β and collagen IV α1, respectively. Of particular interest within the *LamininB1* group are certain hypomorphic mutants that feature prominent defects in cardiac morphogenesis and cardiac ECM layer formation, but in contrast to amorphic mutants, only mild defects in other tissues. All of these alleles carry clustered missense mutations in the laminin LN domain. The identified *Cg25C* mutants display weaker and largely temperature-sensitive phenotypes that result from glycine substitutions in different Gly-X-Y repeats of the triple helix-forming domain. While initial basement membrane assembly is not abolished in *Cg25C* mutants, incorporation of perlecan is impaired and intracellular accumulation of perlecan as well as the collagen IV α2 chain is detected during late embryogenesis.

**Conclusions:**

Assembly of the cardiac ECM depends primarily on laminin, whereas collagen IV is needed for stabilization. Our data underscore the importance of a correctly assembled ECM particularly for the development of cardiac tissues and their lateral connections. The mutational analysis suggests that the β6/β3/β8 interface of the laminin β LN domain is highly critical for formation of contiguous cardiac ECM layers. Certain mutations in the collagen IV triple helix-forming domain may exert a semi-dominant effect leading to an overall weakening of ECM structures as well as intracellular accumulation of collagen and other molecules, thus paralleling observations made in other organisms and in connection with collagen-related diseases.

## Background

Due to its relatively simple cardiac structure, ease of genetic manipulation and conservation of many aspects of cardiac development *Drosophila* is widely used to study specification of cardiac progenitors, cardiomyocyte diversification and differentiation, tubular lumen formation, and as a model for cardiomyopathies (reviewed in [1-5]). The heart (dorsal vessel) of *Drosophila* is a linear tube that pumps hemolymph from the posterior of the abdomen towards the head region from where it returns through an open circulatory system. The dorsal vessel is formed in the embryo from bilateral rows of cardioblasts/cardiomyocytes that merge at the dorsal midline where they undergo defined shape changes to form a contractile heart tube with a wide lumen in the posterior ventricular portion (often referred to as the heart) and a narrower lumen in the anterior region (the aorta). Lumen formation has been shown to require adhesion molecules like DE-cadherin localized in the junctional (J) domains of cardiomyocytes as well as molecules of the Slit/Robo signaling cascade and dystroglycan (Dg) that define an adhesion-free zone in the prospective luminal area, called L-domain [6-9]. Regulation of the width of the lumen appears to involve the collagen XV/XVIII-like molecule multiplexin (Mp), which is specifically expressed in the wider heart portion [10].

Within the dorsal vessel several functionally distinct cell types are discriminated on the basis of the expression of particular transcription factors and morphological features [11-16]: The myocardial tube contains two types of cardiomyocytes (CMs), which are positive for Mef2 and either Tinman (Tin) (in Tin-CMs) or Dorsocross (Doc) and Seven-up (Svp) (in ostial/Svp-CMs that form openings for the inflow of hemolymph in the ventricular heart portion). The myocardial tube is flanked by several types of Mef2-negative non-contractile nephrocyte-like pericardial cells (PCs), which are characterized by expression of *Zfh-1* and either *odd-skipped* (Odd-PCs), *tin* (Tin-PCs) or *tin* plus *even-skipped* (*eve*) (Eve-PCs). All cell types in the dorsal vessel share expression of the *Hand* gene [17]. Specialized multinucleated muscles fibers, the so-called alary muscles (AMs), connect the dorsal vessel with the epidermis. In the embryo there are seven segmental pairs of *org-1* expressing AMs stretching from the apodemes of the lateral epidermis to the dorsal vessel where they surround PCs, preferentially particular Odd-PCs, via delta-shaped extensions [11,18,19]. The dorsal vessel is embedded in an elaborated extracellular matrix (ECM) that is structurally linked to the AMs [20]. The lumen and the outer (abluminal) side of the myocardial tube as well as the PCs are covered by a basement membrane (BM) [11,20,21]. An almost unique component of the cardiac ECM is pericardin (Prc), a collagen-like molecule that is secreted mainly by PCs, Svp-CMs as well as the larval fat body and incorporated into the abluminal ECM [22,23].

While many efforts have been made to complete our knowledge about *Drosophila* cardiogenesis a number of open questions remain. In particular, little is known about genes that guide cardiac morphogenesis or structural components that ensure proper heart function, stability and linkage to other tissues. The specific requirements for AM formation and attachment are also not fully understood. One largely unbiased approach to identify genes required for these processes is chemically induced mutagenesis. We have performed a forward genetic screen for chromosome 2 using EMS as a mutagen and a set of cell type-specific GFP (Green Fluorescent Protein) and RFP (Red Fluorescent Protein) reporters to analyze the development of the embryonic dorsal vessel and its associated alary muscles in parallel with other muscle types. Here we present the characteristics of mutants from two identified complementation groups showing similar phenotypes, namely embryonic AM detachment and dissociation of PCs, as a predominant feature. In both cases defects are caused by domain-specific point mutations in components of the ECM. The members of the first group were found to contain hypomorphic mutations in *Laminin B1* (*LanB1*) encoding the only β chain of *Drosophila* laminins, whereas in the second complementation group the gene *Cg25C* was affected. *Cg25C* (*Dcg1, col4a1*) encodes one of the two type IV collagen chains present in *Drosophila*; the other is encoded by the neighboring gene *viking* (*vkg*, *col4a2*) [24,25]. Although *Drosophila* collagen IV was studied in several developmental contexts, no cardiac-related phenotype has been reported for collagen IV α genes in *Drosophila* prior to this work. Collagens are secreted as homo- or heterotrimeric protomers, with genuine type IV collagens predominantly if not exclusively being found as heterotrimers [26,27]. Based on the conservation of at least two collagen IV genes in diverse organisms such as *Drosophila*, the nematode *Caenorhabditis elegans* and humans as well as on genetic data it is assumed that *Drosophila* collagen IV is also a heterotrimer consisting of two *Cg25C*-encoded α1 chains and one α2 chain encoded by *vkg*, analogous to the most common vertebrate type IV collagen α1_2_α2_1_[28-30]. Laminins are secreted as heterotrimeric glycoproteins and formed by dimerization of one β chain and one γ chain, followed by addition of an α chain [31-33]. In addition to *LanB1*, the *Drosophila* genome encodes a single laminin γ gene, *LanB2*, and two laminin α genes, *Laminin A* (*LanA*) and *wing blister* (*wb*), which are related to vertebrate α3/5 and α1/2 and form the heterotrimers LamininA and LamininW, respectively [34-37].

Together with nidogen and perlecan (in *Drosophila* encoded by *Ndg* and *terribly reduced optic lobes*, *trol*, respectively), laminin and type IV collagen constitute the core components of BMs in all bilaterian organisms, and some of their cell surface receptors such as integrins and dystroglycan are also conserved [38-40]. BMs contribute to the normal differentiation, compartmentalization and integrity of many tissues, and certain mutations in their components have been reported in connection with human diseases (reviewed in [41-43]). In *Drosophila* mutations in laminin-encoding genes have been shown to cause pleiotropic defects in several BM covered organs, including the dorsal vessel, gut, somatic body wall muscles, renal (Malpighian) tubules, trachea, nervous system and wing epithelia [6,37,44-46]. We have isolated novel missense alleles of *LanB1* that have rather limited effects on general embryonic development, but lead to severe defects in the cardiac ECM. Furthermore we report collagen IV mutations that result in poor incorporation of perlecan into BMs, prominent accumulation of intracellular Vkg, and late embryonic cardiac AM detachment. The data presented herein underscore the importance of laminin and collagen particularly for the development of cardiac tissues and their lateral connections. Our work also identifies amino acid residues critical for this function.

## Results

**Figure 1:**
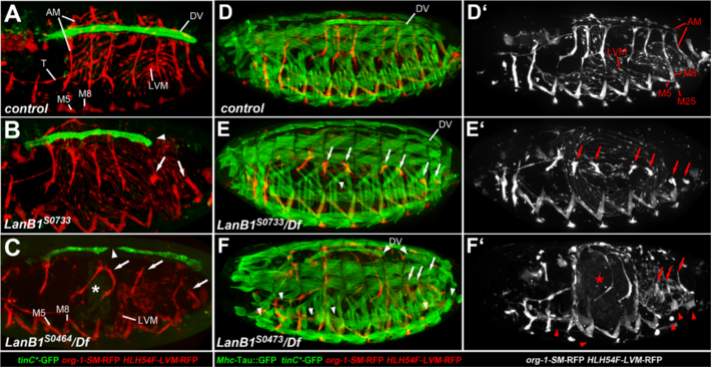
**Novel EMS-induced *LanB1 *alleles associated with alary muscle detachment and abnormal dorsal vessel morphology.** Live stage 16–17 wild-type (*S-18a-13b-16b.1* control) or *LanB1* mutant embryos (dorso-lateral views) carrying tinC*-GFP, *org-1-SM-RFP*, and *HLH54F-LVM-RFP* to monitor the morphology of the dorsal vessel (DV), alary muscles (AM) and longitudinal visceral muscles (LVM), respectively. tinC*-GFP is visible in all cardiomyocytes and weakly in some pericardial cells. RFP is also present in somatic muscles 5, 8 and 25 (partially in focus; M5, M8, M25), and in additional thoracic muscles. Embryos in D-F also carry *Mhc-tau::GFP* for the visualization of the entire musculature (single RFP channel on the right). **(A)** In the wild type, seven pairs of AMs connect to the DV. A pair of anterior longitudinal muscles (thoracic alary-related muscle, T) connects to the midgut. **(B)** Homozygous *LanB1*^*S0733*^ embryo in which the AMs have begun to separate from the DV or are already completely detached (arrows). The DV has retracted from the posterior end (arrowhead). The LVM and thoracic alary-related muscle attachment appear mostly normal. **(C)** Strong alleles such as *LanB1*^*S0464*^ (here over the *LanB1*-deleting deficiency *Df(2L)ED12527*) show a more pleiotropic phenotype, in which the midgut (*) lacks constrictions and is only partially associated with visceral musculature. Severe heart defects such as gaps (large arrowhead) are more frequent than in *LanB1*^*S0733*^. **(D, D′)** Control embryo with additional *Mhc-tau::GFP* showing the wild-type muscle pattern. **(E, E′)***LanB1*^*S0733*^*/Df(2L)ED12527* embryo. The body wall musculature shows only minor detachment or loss of a few fibers (small arrowhead) while most AMs are already detached at this time point (arrows). **(F, F′)** Amorphic *LanB1*^*S0473*^ allele over *Df(2L)ED12527*. In addition to AMs many fibers of the body wall musculature detach resulting in an irregular shape or gaps (small arrowheads). Furthermore the visceral musculature fails to surround the unconstricted ball-shaped midgut.

We have screened EMS-induced 2^nd^ chromosome mutants for defects in cardiac, somatic and visceral muscles. The mutagenesis was performed in flies carrying tinC*-GFP, *org-1-SM-RFP* and *HLH54F-LVM-RFP* reporter genes in order to visualize cardioblasts of the embryonic dorsal vessel, Org-1-positive somatic muscles, and longitudinal visceral muscles (LVM), respectively. The somatic muscles labeled by *org-1-SM-RFP* include seven pairs of alary muscles (AMs) that are connected with the dorsal vessel via delta-shaped attachments (Figure 1A). Mutants were divided into several phenotypic classes based on the information obtained from the three reporters. Here we focus on a phenotypic class that is characterized by detachment of AMs from the dorsal vessel as its primary feature. Further genetic analysis allowed us to allocate most of these mutants to two genetically interacting complementation groups that correspond to the laminin β chain-encoding gene *LanB1* or the type IV collagen-encoding gene *Cg25C* (see “Methods” for genetic mapping procedures with these mutations).

### Isolation of novel EMS-induced *LanB1* alleles

Three of the newly isolated *LanB1* mutants, *S0733*, *S1163* and *S3773*, are characterized by detached AMs and an irregular morphology of the dorsal vessel, while muscle fibers in the body wall musculature show only mild defects and visceral muscles are barely affected (Figure 1B,E,E′; compare to Figure 1A,D,D′ respectively; see also Additional file 1: Figure S1A-C). In most cases detached AMs are first observed at the posterior portion of the heart of stage 16–17 embryos. Prior to hatching, these embryos usually lose dorsal attachment of almost all AMs except for the anterior-most pair that anchors near the lymph gland (Figure 1E,E′). *Org-1-*RFP-labeled, alary-related muscles stretching longitudinally through the thorax also maintain anchorage in most embryos. Defects in the cardiac tube itself such as twists, stretches of single-rowed cardiomyocyte alignments and occasional breaks are observed increasingly during late development. Just prior to eclosion the heart tube appears to lose its anchorage at the posterior end, the ventricle collapses and the entire tube shortens significantly (see also time-lapse studies described below). The described features are present in homozygous embryos of the three lines and in embryos with trans-heterozygous allele combinations (Figure 1B,E,E′; Additional file 1: Figure S1B, C and data not shown).

This relatively specific phenotype, affecting mainly alary muscles and the morphology of the heart tube, is more restricted than the phenotype of strong *LanB1* alleles also isolated in our screen (*LanB1*^*S0212*^, *LanB1*^*S0464*^, *LanB1*^*S0473*^, *LanB1*^*S1522*^, *LanB1*^*S2941*^). The latter also show alary muscle and dorsal vessel defects, but were put into a distinct phenotypic class mainly because of their severe midgut constriction defects and visceral mesoderm phenotypes (Figure 1C,F,F′; Additional file 1: Figure S1D, E and data not shown). LVM fibers can be found in bundles (together with circular visceral muscles) loosely flanking the unconstricted midgut. *LanB1* mutants of this group also have more pronounced defects in the heart (arrowheads in Figure 1C,F) and in somatic body wall muscles (e.g., small arrowheads in Figure 1F, compare with Figure 1D,E). Altogether the phenotype of the strong *LanB1* alleles resembles that of previously reported *LanB1* null mutants [46] and amorphic mutants for the laminin γ chain-encoding *LanB2* gene (Additional file 1: Figure S1F and [45]). In mutants for the two laminin α chain genes *wb* (*α1,2*) and *LanA* (*α3,5*) the same tissues were affected, but milder phenotypes were seen for alary muscles and the LVM, respectively (Additional file 1: Figure S1G-J). Our EMS screen generated at least eight very similarly looking *wb* alleles, thus both laminin genes located on chromosome 2 were hit with similar frequency. The most conspicuous features of *wb* mutants were, in accordance with previous descriptions, abnormal midgut morphology (partial lack of constrictions), variably detached LVM fibers and a partially interrupted myocardial tube (Additional file 1: Figure S1G,H; [37,45]). Unlike in the strong and weak *LanB1* mutants described above, AM attachment is largely maintained and a recognizable heart lumen appears to be present. In contrast, amorphic *LanA* mutants analyzed with our GFP/RFP marker set exhibit a *LanB1*-like myocardial phenotype with similar AM detachment, but no or very mild midgut and LVM defects (Additional file 1: Figure S1I, J; [44]).

### Hypomorphic *LanB1* mutants with heart defects harbor domain-specific missense mutations

**Figure 2:**
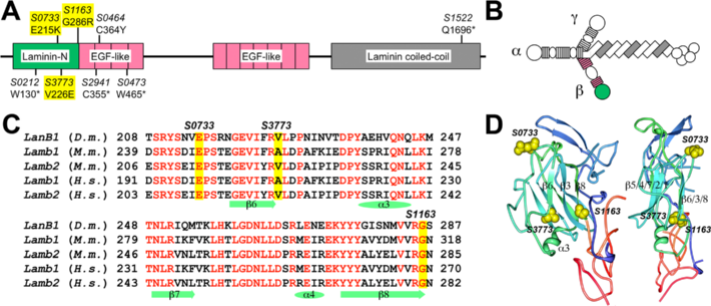
**Allocation of EMS-induced *LanB1 *mutations. (A)** Domain organization of *Drosophila* Laminin B1 and location of the isolated mutations. The affected amino acid positions with the corresponding change are given below the allele number (* = stop). The described hypomorphic *LanB1* mutations (yellow) all cluster within the Laminin N (LN) domain. **(B)** Schematic of the laminin α/β/γ trimer illustrating the location of the β laminin domains using the color-coding shown in A. **(C)** Alignment of the C-terminal part of the LN domain from *Drosophila melanogaster* LanB1 (D.m., GenBank AAF52563) with that of β laminins Lamb1 and Lamb2 of mouse (M.m., AAI50810.1, AAC53535.1) and human (H.s., AAA59482.1, AAB34682.2). Red letters indicate 100% identity. Secondary structure elements below are according to the structure model shown in D. **(D)** 3D-structure of mouse Laminin β1 LN-LE_1_ (4AQS, [47], illustrated using the Protein Workshop tool [48]). The LN domain (blue/green) and the closely linked first EGF-like (LE) domain (red) are depicted from two angles. The amino acid residues that correspond to those affected in the hypomorphic *LanB1* alleles (highlighted in yellow) are situated on the same side of the β-sandwich structure.

Given the distinct phenotypes present in hypomorphic versus strong *LanB1* alleles we asked whether specific types of mutations are present in each phenotypic class. Sequencing of DNA from homozygous embryos showed that this is indeed the case. The domain organization of the laminin β chain and the positions of the determined mutations are shown in Figure 2A, next to a schematic illustrating the location of the domains within a prototype laminin α/β/γ trimer (Figure 2B). Strikingly, all hypomorphic *LanB1* alleles isolated on the basis of a largely heart-restricted phenotype carry point mutations that cluster within the N-terminal LN domain (Figure 2A). In *LanB1*^*S0733*^ an EMS-typical G-to-A transition in codon 215 converts an acidic glutamate residue to the basic lysine. In *LanB1*^*S1163*^ a G-to-A exchange results in a glycine-to-arginine switch at codon 286. Both of these detected amino acid substitutions change conserved residues (see partial alignment in Figure 2C). In *LanB1*^*S3773*^ a T-to-A transversion changes a conserved valine to glutamate in position 226. Heteroallelic combinations involving *LanB1*^*S3773*^ display slightly milder phenotypes, in which the posterior end of the heart tube remains frequently attached and escapers survive up to early pupal stages. By contrast, *LanB1*^*S0733*^ and *LanB1*^*S1163*^ mutants or their trans-allelic combinations die during the early first instar.

The high sequence conservation of the LN domain between *Drosophila* and mammals makes it possible to superimpose the affected positions to the known structure of the LN domain of mouse LanB1. The LN domain contains a core of eight β-strands folded into two sheets of a sandwich structure [47]. All of the identified changes are located on the same side of this β-sandwich, potentially disturbing the structure of the β6/β3/β8 interface (Figure 2D). Because of their common features and for brevity we refer to these hypomorphic *LanB1* alleles in the following as *LanB1*^*LN*^ mutants.

In contrast to the *LanB1*^*LN*^ mutants, the strong and more pleiotropic phenotypes are mostly associated with premature stop codons in *LanB1*. We found truncations that occur either early within the laminin chain, as in the alleles *LanB1*^*S0212*^, *LanB1*^*S2941*^ and *LanB1*^*S0473*^, or late near the C-terminus, as in *LanB1*^*S1522*^ (Figure 2A). We do not see significant phenotypic differences between these truncated alleles in either homozygous or hemizygous conditions (Figure 1F, F′; Additional file 1: Figure S1D, E and data not shown), which we attribute to the absolute requirement of C-terminal portions for trimer formation and laminin secretion. All of these truncated products would lack a cysteine near the C-terminal end of the coiled-coil region that was shown to form a disulfide bridge with the corresponding region of the laminin γ chain [49,50]. These alleles are therefore most likely functional null alleles. Notably, exchange of an intra-molecular disulfide bridge-forming cysteine residue in position 364 in the second EGF-like repeat to tyrosine also appears to have a severe impact on the functionality of laminin, since *LanB1*^*S0464*^ mutants (either homozygous or in combination with a *LanB1*-deleting deficiency) show strong phenotypes similar to those with LanB1 truncations (Figure 1C, compare with Figure 1F, F′ and Additional file 1: Figure S1D, E).

### Mutations in the collagen IV-encoding gene *Cg25C* disturb the connection of alary muscles with the dorsal vessel

**Figure 3:**
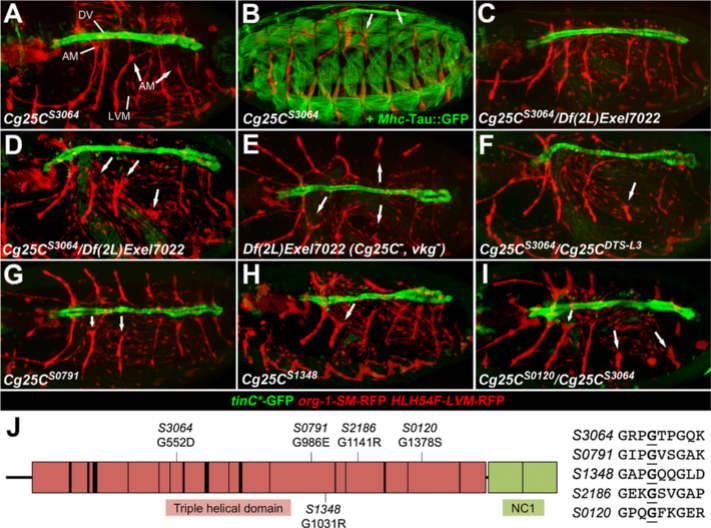
**Alary muscle attachment defects caused by EMS-induced mutations in the collagen IV-encoding gene *Cg25C*. (A-I)** Live preparations of *Cg25C* mutant embryos with GFP and RFP markers as in the corresponding control in Figure 1A and raised at 25°C. The embryo in panel B additionally carries *Mhc-tau::GFP* as the corresponding control in Figure 1D. **(A)** Homozygous *Cg25C*^*S3064*^ embryo at early stage 17 in which some alary muscles (AMs, arrows) start to detach from the dorsal vessel. The LVM is normal. **(B)** The body wall musculature develops mostly normally in homozygous *Cg25C*^*S3064*^ mutants. **(C)** Hemizygous *Cg25C*^*S3064*^ embryo with the *Cg25C*-deleting deficiency *Df(2L)Exel7022* at early stage 17 as **(A)**, but with AMs still attached to the dorsal vessel. **(D)***Cg25C*^*S3064*^/*Df(2L)Exel7022* embryo at late stage 17, in which many AMs, particularly in the middle portion, lose their dorsal vessel attachment. **(E)** Late stage 17 embryo homozygous for *Df(2L)Exel7022*, in which both collagen IV-genes, *Cg25C* and *vkg*, are deleted, shows no enhancement in the detachment phenotype as compared to *Cg25C*^*S3064*^ homo- and hemizygotes. **(F)** AM detachment is also apparent in the majority of stage 17 embryos of the genotype *Cg25C*^*DTS-L3*^*/Cg25C*^*S3064*^. A milder detachment phenotype is observed in a fraction of late stage 17 embryos for the alleles *Cg25C*^*S0791*^**(G)** and *Cg25C*^*S1348*^**(H). (I)** Alary muscle detachment also occurs in the combination *Cg25C*^*S0120*^*/Cg25C*^*S3064*^. **(J)** Schematic of *Cg25C*-encoded collagen IV illustrating the positions of the mutations found in the indicated EMS alleles. The collagen triple helix-forming domain (red) consists of Gly-X-Y repeats that are interrupted at positions marked by vertical bars. All mutations found in this study are missense mutations at the glycine of a Gly-X-Y repeat (underlined in the sequence with flanking repeats shown to the right).

A second complementation group originating from mutants with alary muscle detachment is formed by the alleles *S0120*, *S0791*, *S1348*, *S2186*, and *S3064* of *Cg25C*, which encodes one of the two type IV collagens in *Drosophila*. This complementation group was established through analysis of the line *S3064*, which was isolated based on its fully penetrant alary muscle detachment phenotype in stage 17 embryos (Figure 3A, B and data not shown). The *Cg25C*^*S3064*^ mutant phenotype resembles that of the hypomorphic *LanB1*^*LN*^ alleles except for the fact that detachment usually starts later and predominantly with central rather than posterior alary muscles. In *Cg25C*^*S3064*^*/Df(2L)Exel7022* embryos, alary muscles begin to detach at about the same time as body wall muscles start to contract and in some cases just around hatching time (even though most embryos fail to hatch) (compare the younger embryo of Figure 3C with Figure 3D). Embryos homozygous for the deficiency *Df(2L)Exel7022*, which deletes both of the *Drosophila* collagen IV genes, *Cg25C* and *viking* (*vkg*), have an alary muscle phenotype comparable to that of *Cg25C*^*S3064*^*/Df(2L)Exel7022*, although with lower penetrance (Figure 3E, Table 1). Notably, we also detect alary muscle detachment in homozygotes for the previously reported allele *Cg25C*^*DTS-L3*^ (data not shown; [30]) and in *Cg25C*^*DTS-L3*^*/Cg25C*^*S3064*^ embryos (Figure 3F, Table 1).

**Table 1:** **Frequency of AM detachment phenotypes in *Cg25C *** **mutants**

**Genotype**	**Fraction of abnormal embryo half-sides**		
	**19°C**	**25°C**	**29°C**
*Cg25C*^*S3064*^*/CyO*	n.d.	0% (n = 58)	n.d.
*control/Df(2L)Exel7022*	n.d.	1.5% (n = 66)	1.8% (n = 56)
*Cg25C*^*S0120*^*/Df(2L)Exel7022*	40% (n = 15)	79.6% (n = 142)	73.3% (n = 30)
*Cg25C*^*S0791*^*/Df(2L)Exel7022*	0% (n = 38)	87.1% (n = 62)	93.3% (n = 30)
*Cg25C*^*S1348*^*/Df(2L)Exel7022*	6.5% (n = 31)	76.6% (n = 94)	93.3% (n = 30)
*Cg25C*^*S2186*^*/Df(2L)Exel7022*	3.4% (n = 59)	15.3% (n = 85)	83.3% (n = 30)
*Cg25C*^*S3064*^*/Df(2L)Exel7022*	32.4% (n = 34)	99% (n = 104)	89.2% (n = 74)
*Cg25C*^*DTS-L3*^*/Df(2L)Exel7022*	8.1% (n = 34)	100% (n = 88)	88.2% (n = 76)
*Cg25C*^*S3064*^*/Cg25C*^*DTS-L3*^	10% (n = 30)	100% (n = 72)	100% (n = 75)
*Df(2L)Exel7022/Df(2L)Exel7022*	73.3% (n = 15)	54% (n = 87)	63.5% (n = 63)

Embryos were analyzed for the position of AM attachments at late stage 17 (trachea become clearly visible) after development at the indicated temperature. Embryo half-sides in which at least one of the seven AMs showed an increased distance between its dorsal end and the DV were counted as abnormal (n = total number of scored half-sides; control = *S-18a-13b-16b.1* with GFP/RFP reporter constructs; analogous 3rd chromosome reporters were crossed in for analysis of genotypes lacking 2nd chromosome reporters).

The alleles *Cg25C*^*S0791*^ and *Cg25C*^*S1348*^ showed a slightly weaker and not fully penetrant phenotype in homozygous embryos (Figure 3G, H) or in trans-heterozygous embryos containing one copy of the allele over *Df(2L)Exel7022* or *Cg25C*^*S3064*^ at our standard screening temperature of 25°C (Table 1 and data not shown). In addition, the alleles *Cg25C*^*S0120*^ and *Cg25C*^*S2186*^, which in homozygous condition show additional features (likely due to other mutations), were found to be lethal with *Df(2L)Exel7022* and *Cg25C*^*S3064*^ and semi-lethal with *Cg25C*^*k00405*^ (with rare escapers at about 6-9% of the expected rate). While *Cg25C*^*S0120*^ was also lethal with the weaker alleles *Cg25C*^*S0791*^ and *Cg25C*^*S1348*^, the *Cg25C*^*S2186*^ allele shows nearly complete inter-allelic complementation of lethality in these combinations. This fits with a robust alary muscle detachment phenotype in most *Cg25C*^*S0120*^*/Cg25C*^*S3064*^ and *Cg25C*^*S0120*^*/Df(2L)Exel7022* embryos, but rare occurrence in the corresponding trans-allelic *Cg25C*^*S2186*^ embryos at 25°C (Figure 3I, Table 1 and data not shown). Detachment of some alary muscle fibers was however detected in *Cg25C*^*S2186*^*/Cg25C*^*S3064*^ and *Cg25C*^*S2186*^*/Df(2L)Exel7022* embryos at 29°C (Additional file 2: Figure S2C, D, Table 1 and data not shown).

In addition to the (partial) interallelic non-complementation among these *Cg25C* alleles we also observed trans-allelic genetic interactions with *LanB1* alleles. If *Cg25C*^*S3064*^ is crossed with strong *LanB1* alleles only 0-30% of the expected non-Cy siblings eclosed as adults and from crosses with *LanB1*^*LN*^ mutants only 3-40% eclosed. The presence of a second-site mutation in *LanB1* on the *Cg25C*^*S3064*^ mutant chromosome was ruled out as an explanation for these results by sequencing of the *LanB1* locus. Furthermore, a similar or slightly milder reduction of viability was observed in crosses of several *LanB1* EMS alleles with *Cg25C*^*DTS-L3*^ and *Cg25C*^*S0120*^. Although trans-heterozygous embryos and early L1 larvae with *Cg25C* alleles over the *LanB1*-deleting deficiency *Df(2L)ED12527* or *LanB1*^*S0473*^ rarely show any significant detachment phenotypes (when analyzed for tinC*-GFP, *org-1-SM-*RFP and *HLH54F-LVM-*RFP reporters; data not shown), this observed genetic interaction reinforces the notion that *LanB1* and *Cg25C* engage in close functional interactions.

### Temperature-sensitivity of *Cg25C*-related alary muscle detachment phenotypes

Since several collagen alleles including *Cg25C*^*DTS-L3*^ were recently reported to be associated with temperature-sensitivity [30] we reinvestigated the alary muscle phenotype and viability in selected genotypes at different temperatures. As noted before, the detachment phenotype was not fully penetrant for some trans-allelic *Cg25C* combinations at our standard temperature of 25°C or below. In agreement with temperature-sensitivity, the frequency of the detachment phenotype is enhanced for all our alleles (but not for the deficiency) at elevated temperature (Table 1, Additional file 2: Figure S2A-D and data not shown). Furthermore, the combinations *Cg25C*^*DTS-L3*^*/Cg25C*^*S3064*^ and *Cg25C*^*S0120*^*/Cg25C*^*S3064*^ are embryonic lethal at 25°C, but if kept at 19°C some animals develop into early L1 and L2 larvae, respectively. In agreement with the weaker phenotype of *Cg25C*^*S0791*^, *Cg25C*^*S1348*^ and *Cg25C*^*S2186*^, adult escapers were observed for these alleles if combined with *Cg25C*^*S3064*^ (or *Cg25C*^*DTS-L3*^) and grown at 19°C, while at 25°C these trans-heterozygotes died either during larval growth or in pupal stages. These data imply that temperature-sensitivity is a more widespread phenomenon of collagen mutations (see also discussion).

### Molecular identification of EMS-induced mutations in *Cg25C* reveals changes in collagen glycine-X-Y repeats

Sequencing of the loci from the *Cg25C* alleles *S0120*, *S0791*, *S1348*, *S2186* and *S3064* detected an allele-specific point mutation in each of the five cases. As illustrated in Figure 3J all *Cg25C* alleles isolated in our screen contain glycine exchanges originating from G-to-A nucleotide transitions at different positions. Glycine residues are abundant in the collagen triple-helix-forming domain as part of the structurally important Gly-X-Y repeats (in which Y is often proline or hydroxyproline). Surprisingly, *Cg25C*^*S3064*^ shares its glycine-to-aspartic acid mutation at position 552 with the recently published allele *Cg25C*^*DTS-L3*^[30]. This allele was characterized as dominant temperature-sensitive in the context of viability, female fertility and oviduct muscle stability [30]. The proposed antimorphic features possibly explain the relatively strong alary muscle detachment phenotype of G552D homozygous mutants, which is more penetrant than that of complete collagen IV null mutants (*Df(2L)Exel7022*) at 25-29°C (see Table 1). In another strong allele, *Cg25C*^*S0120*^, the glycine at residue 1378 is converted into a serine. The milder allele *Cg25C*^*S0791*^ contains a glycine-to-glutamic acid change at residue 986. A glycine-to-arginine exchange is found at amino acid positions 1031 and 1141 of the relatively weak alleles *Cg25C*^*S1348*^ and *Cg25C*^*S2186*^, respectively.

### Alary muscle detachment in *LanB1*^*LN*^ and *Cg25C* mutants is connected with dissociation of pericardial cells from the dorsal vessel

**Figure 4:**
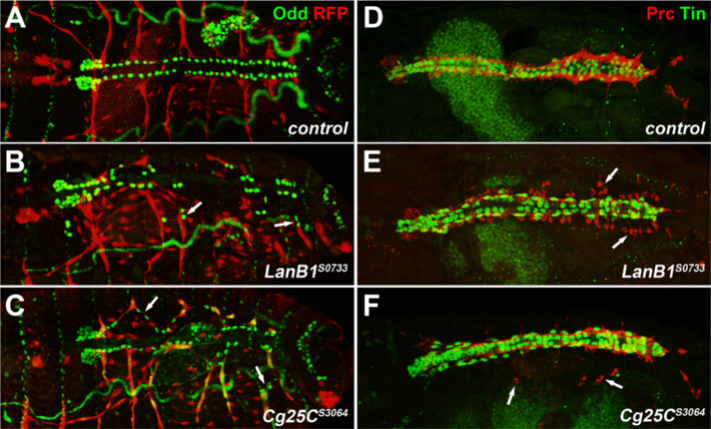
**Joint detachment of alary muscles and Odd-positive pericardial cells in *LanB1 *and *Cg25C *mutants.** Dorsal views of wild-type and *LanB1*^*S0733*^ and *Cg25C*^*S3064*^ mutant embryos at stage 16/17 stained for Odd (green) and RFP expressed from the reporter genes as in Figure 1 (red) **(A-C)** or stained for Prc (red) and Tin (green) **(D-F)**. **(A)** Control embryo with the dorsal ends of all seven pairs of alary muscles terminating near the two rows of Odd-PCs. **(B)***LanB1*^*S0733*^ embryo with detached AMs ending near dislocated PCs (arrows). **(C)** A similar phenotype is detected in *Cg25C*^*S3064*^ embryos. **(D)** Control embryo showing normal arrangement of Tin^+^ myocardial and pericardial cells and a contiguous distribution of Prc flanking the outside of the dorsal vessel. **(E)***LanB1*^*S0733*^ embryo with irregular Prc distribution often at some distance to the heart (arrows). **(F)***Cg25C*^*S3064*^ embryo with contiguous Prc distribution, but some of the pericardial Prc signals are found in laterally located patches (arrows). Tin-positive PCs are associated with the dorsal vessel in both mutants **(E, F)**.

Alary muscles attach to the dorsal vessel through ECM fibers surrounding PCs [19,20] and mutations in the laminin α and γ chain-encoding genes *LanA*, *wb*, and *LanB2* have previously shown to impair cardiac attachment of PCs [37,44,45]. These data together with initial hints obtained with the tinC*-GFP marker suggested that alary muscles detach together with PCs in *Cg25C* and *LanB1*^*LN*^ mutants. Juxtaposition of detached dorsal alary muscle endings with PCs could indeed be detected in *Cg25C* and *LanB1*^*LN*^ mutants by double-staining of *org-1*-RFP and the PC marker Odd (Figure 4A-C). In contrast, Tin-positive PCs were still detectable at their normal position close to cardiomyocytes in both of these mutants at a similar stage (Figure 4D-F). Pericardin (Prc), a collagen-like molecule secreted in embryos mainly by PCs and normally deposited at the abluminal side of the heart tube (Figure 4D) was also dislocated in those mutants (e.g., Figure 4E). Furthermore, Prc did not form a contiguous layer around the dorsal vessel suggesting severe problems in the establishment of cardiac ECM structure in the isolated hypomorphic *LanB1*^*LN*^ alleles similar to those observed in *LanB1* null mutants [46]. In contrast, no discontinuity in the Prc distribution was observed in *Cg25C*^*S3064*^ embryos except for some lateral spots, which indicate the presence of detached PCs (Figure 4F).

**Figure 5:**
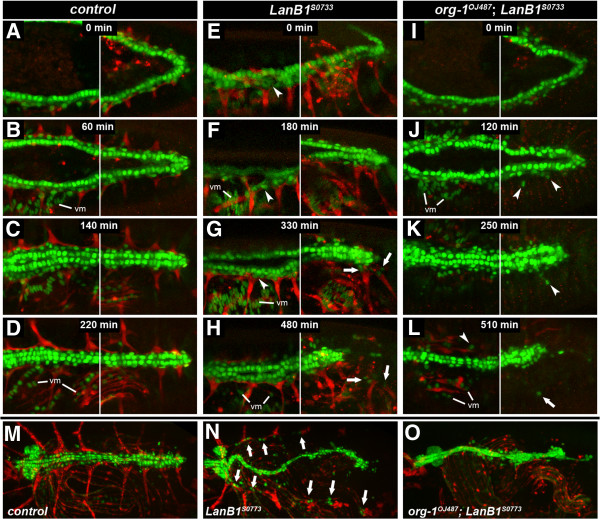
**Live imaging of alary muscle and pericardial cell migration during dorsal closure.** Time-lapse images (dorso-lateral views) from stages 15 to 17 embryos **(A-L)** and still image of another late stage 17 embryo (M-O) carrying *Hand-GFP* in addition to the reporters explained in Figure 1. *Hand*-GFP labels cardioblasts and PCs, and is also active in the nuclei of the circular visceral musculature (partially in focus, labeled vm). Relative time points are indicated at the upper center. (**A-D**, Additional file 3: Movie S1, **M**) Control embryo. AMs labeled by *org-1*-RFP and PCs expressing *Hand*-GFP are in close contact with cardioblasts throughout dorsal closure and during fast movements at the end of embryogenesis. (**E-H**, Additional file 4: Movie S2, **N**) Homozygous *LanB1*^*S0733*^ embryo (longer total imaging time due to slower development). **(E, F)** At stage 15 most heart cells migrate normally, but single PCs are already lagging behind cardioblasts (arrowheads). The AMs are still attached close to the PCs. **(G)** Around the time of heart tube closure the posterior AMs start to detach from the heart (arrows). **(H)** The dorsal vessel shortens and the distance to AMs grows. As they contract AMs pull away individual or small groups of PCs (arrows), which is also seen with more anterior AMs in all analyzed late stage 17 embryos prior to hatching **(N)**. (**I-L**, Additional file 6: Movie S4, **O**) Alary muscle-deficient double mutant *org-1*^*OJ487*^; *LanB1*^*S0733*^. **(I)** Stage 15 embryo with correctly specified heart cells but without AM fibers essentially as in *org-1* single mutants (not shown). **(J, K, L)** During dorsal closure some of the PCs are located at intermediate distance to cardioblasts (arrowheads) similar to *LanB1*^*S0733*^ single mutants, but extreme displacement towards the lateral is only rarely observed for single PCs (arrow in **L**) or not seen at all **(O)**.

In principle wrong positioning of alary muscle endings and PCs could be a consequence of defects in cell migration or PC arrangement. Time-lapse studies were performed to address this possibility and to visualize the order of events. An additional reporter, *Hand*(*HCH)-*GFP [51], was crossed in for parallel live observation of PCs and alary muscles. In the wild type, dorsal alary muscle endings are clearly visible next to developing PCs and near cardioblasts from stage 14 onwards (Figure 5A-D and data not shown). Dorsal alary muscle extensions and PCs closely follow the migration of cardioblast rows towards the dorsal midline (Figure 5A-D, Additional file 3: Movie S1). This is not always the case in *LanB1*^*S0733*^ mutants where some PCs can be found lagging behind migrating cardioblasts (Figure 5E,F, Additional file 4: Movie S2). As contraction of somatic and cardiac muscles initiates, the heart tube loses anchorage and larger gaps between cardiomyocytes and PCs appear, most often beginning from the posterior as described above for alary muscle detachment. Eventually single or small groups of PCs are pulled towards lateral locations by retracting alary muscles in all late stage 17 *LanB1*^*S0733*^ mutant embryos (n = 20; Figure 5G,H,N; Additional file 4: Movies S2, Additional file 5: S3). These data imply that the contracting alary muscles actively contribute to PC dislocation. In order to prove this further we performed similar experiments with double mutants that lack alary muscles. Embryos with a null mutation in the *org-1* gene fail to generate any alary muscles [18]. In *LanB1*^*S0733*^ embryos that are also mutant for *org-1* only a small number of lagging PCs was detected during dorsal closure and some PCs were mildly detached after heart tube formation. Unlike in *LanB1*^*LN*^ single mutants, extreme dislocation of PCs toward the lateral occurs rarely and then only with single cells in the double mutant (Figure 5I-L,O; Additional file 6: Movie S4). Similar observations were made in *Cg25C*^*S3064*^*org-1* double mutants with the difference that short distance PC detachment was limited to very late stages (data not shown).

Taken together these data demonstrate that the close association of pericardial cells with the myocardial tube is weakened in the isolated mutants and as muscle contraction begins pericardial cells contacted by alary muscles eventually get pulled away.

### Cardiomyocyte arrangement and polarity in *LanB1*^*LN*^ and *Cg25C* mutants

**Figure 6:**
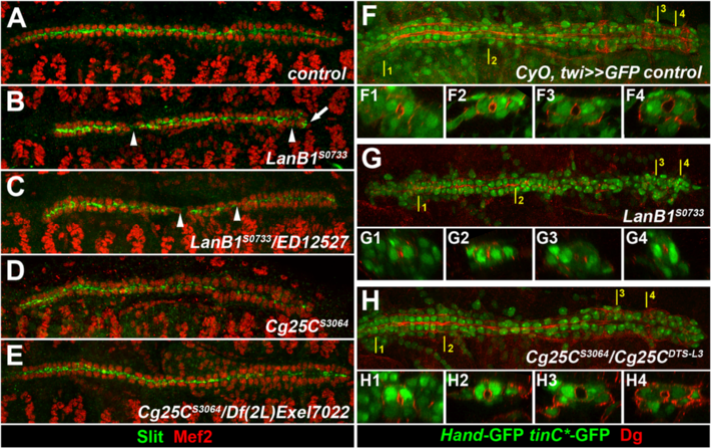
**Analysis of cardiomyocyte arrangement and polarity. (A-E)** Dorsal views of stage 17 embryos stained with antibodies against Mef2 and Slit as indicated. **(A)** Normal arrangement of cardiomyocytes in two rows with Slit localization predominantly at the luminal side. In homozygous *LanB1*^*S0733*^**(B)** and hemizygous *LanB1*^*S0733*^/*Df(2L)ED12527***(C)** embryos cardiomyocytes are arranged more densely, but mostly still with normal polarity. Small gaps and stretches with alignment defects are observed leading to occasional interruptions (arrowheads) or mislocalization of Slit (arrow) at these positions. **(D)** In *Cg25C*^*S3064*^ and in *Cg25C*^*S3064*^/*Df(2L)Exel7022***(E)** embryos cardiomyocytes align essentially as in wild type with normal luminal Slit localization except for a slightly weaker Slit concentration in the posterior heart portion. Minor cardioblast misalignments in the posterior heart are observed only in *Cg25C*^*S3064*^ homozygotes. **(F-H)** Stage 17 embryos with a *Hand-*GFP transgene to visualize cardiomyocytes and PCs, co-stained with antibodies against Dystroglycan (Dg). YZ plane cross-sections are shown below each XY projection at the indicated positions. **(F)** Heterozygous *Cg25C* embryos as identified by the presence of balancer encoded twi-GAL4/UAS-GFP fluorescence showing normal Dg localization at the luminal and outer membrane domains of cardiomyocytes. A large lumen is formed in the ventricle (F3, F4) and a narrower one in the aorta (F1, F2). **(G)** Dorsal vessel of a *LanB1*^*S0733*^ embryo with interrupted heart cell alignment and discontinuous Dg staining especially at its outer surface and in the ventricular portion. Along the anterior-posterior axis sections containing no lumen or a small lumen are present (G1-4). (**H**, H1-4) Embryo with the homozygous G552D mutation in the *Cg25C*^*S3064*^*/Cg25C*^*DTS-L3*^ background shows normal Dg localization and lumen formation.

**Figure 7:**
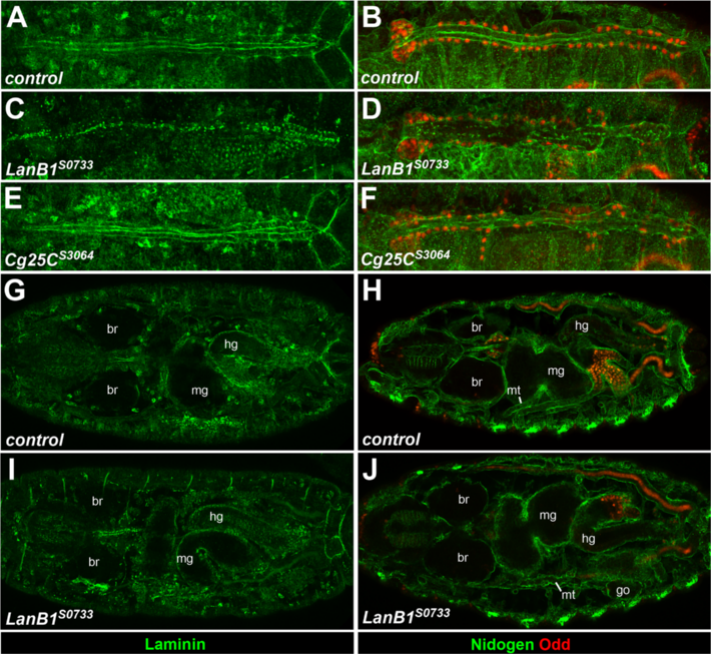
**Distribution of the ECM components laminin and nidogen in *LanB1***^***S0733***^**and *Cg25C***^***S3064***^**mutant embryos.** Dorsal views of stage 16–17 embryos stained with antibodies against laminin **(A, C, E, G, I)** or nidogen and Odd **(B, D, F, H, J)** as indicated. **(A, B)** Dorsal vessel of a wild type embryo with contiguous laminin and nidogen layers in the lumen and around the myocardial tube. **(C, D)** Dorsal vessel of a *LanB1*^*S0733*^ embryo displaying only short stretches of laminin and nidogen deposits and numerous dots or small circles scattered relatively evenly along the tube at about one dot per cardiomyocyte. **(E, F)***Cg25C*^*S3064*^ mutant with nearly normal laminin and nidogen distribution. **(G, H)** Projection of internal sections showing laminin **(G)** and nidogen **(H)** staining in basement membranes of other tissues, e.g. around the brain (br), gonad (go), hindgut (hg), midgut (mg) and Malpighian tubules (mt).

The data imply that the properties of the outer surface of the cardiac tube are critical for PC and alary muscle attachment. Hence, the way cardiomyocytes are oriented within the heart tube could be a contributing factor to the observed effects. To get more information about the arrangement and the polarity of cardiomyocytes in *LanB1*^*LN*^ and *Cg25C* mutants we performed immunostainings for the myocardial transcription factor Mef2, the guidance molecule and polarity marker Slit, and the ECM receptor dystroglycan (Dg). Slit and Dg are critical for lumen formation and normally are concentrated at the luminal domain of cardiomyocytes, Dg is also present at the abluminal membrane domain, but both molecules are absent from junctional domains of cardiomyocytes (Figure 6A,F; [7,9,52,53]).

As already noticed in our observations with tinC*-GFP and *Hand*-GFP, *LanB1*^*LN*^ mutants feature occasional gaps or single-rowed stretches of Mef2-stained cardiomyocytes (arrowheads in Figure 6B,C) that are accompanied by denser spacing of cells in the remainder of the dorsal vessel. *Cg25C* mutants show almost normal cardiomyocyte alignment. The frequent misalignment of few cardiomyocytes in the posterior heart of *Cg25C*^*S3064*^ homozygotes (e.g. in Figure 6D) is unlikely to result from the *Cg25C*^*S3064*^ mutation alone, as it is rarely found in *Cg25C*^*DTS-L3*^ or trans-allelic *Cg25C*^*S3064*^ combinations (Figure 6E, see also Figure 3).

Slit was detected at the luminal side along most of the cardiac tube in *LanB1*^*S0773*^ and *Cg25C*^*S3064*^ mutants, indicating that polarization of cardiomyocytes is not generally disturbed. However, in *LanB1*^*LN*^ mutants the luminal Slit layer appears to be less regular and interruptions and dislocated patches are observed at positions that show breaks in the cardiac alignment (Figure 6B, C). The condensed arrangement of posterior cardiomyocytes and irregular Slit localization suggests that formation of a ventricle with a wide lumen does not occur in these mutants, which is confirmed in co-stainings of *Hand*-GFP with anti-Dg antibodies and corresponding luminal cross-sections (Figure 6G, G1-4, compare to Figure 6F, F1-4). Areas forming a small lumen alternate with collapsed portions in both parts of the dorsal vessel, ventricle and aorta. Furthermore, Dg localization at the luminal and even more frequently at the outer domain is interrupted along the anterior-posterior axis in *LanB1*^*S0773*^ mutants (Figure 6G) suggesting that laminin contributes to Dg stabilization.

*Cg25C* mutants essentially look like wild type in Mef2/Slit and Dg stainings, except that the Slit signal in the ventricular portion is even less robust than in the wild type (Figure 6D,E). Interestingly, multiplexin, a collagen XV/XVIII-like molecule specifically expressed in the ventricle, has recently been shown to affect ventricular heart lumen formation by enhancing myocardial Slit/Robo signaling [10]. However, the level of ventricular Slit in type IV collagen mutants appears to be sufficient for normal lumen formation (Figure 6H and live observations not shown; see also Figure 3D-I).

Altogether our data show that even hypomorphic mutations in the laminin β chain can affect the stable arrangement of cardiac cells and lumen formation in the dorsal vessel. In contrast, they do not support a specific function for type IV collagen during establishment of cell polarity or initial steps of lumen formation, although this does not exclude a later function in their maintenance.

### A discontinuous ECM layer is formed around the cardiac tube in *LanB1*^*LN*^ mutants

**Figure 8:**
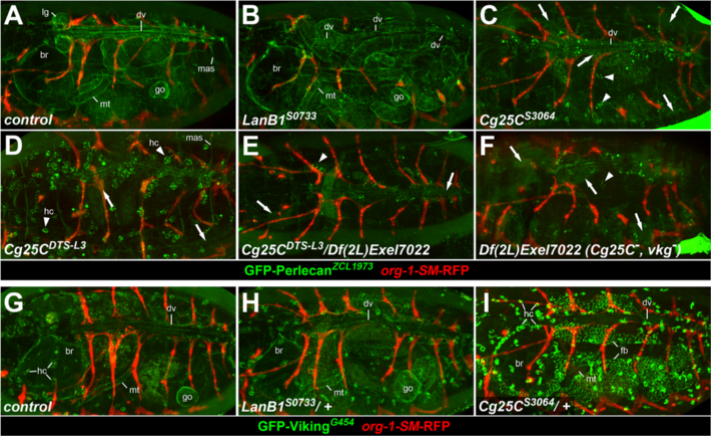
**The collagen glycine repeat mutation G552D causes abnormal distribution of perlecan and Col4a2/Viking.** Live fluorescent images of stage 17 embryos carrying GFP protein trap insertions *trol*^*ZCL1973*^ or *vkg*^*G454*^ to analyze the distribution of perlecan **(A-F)** or Vkg/Col4a2 **(G-I)**, respectively. The *org-1-SM-RFP* reporter gene drives cytoplasmic RFP expression in *org-1*-positive somatic muscles as in Figure 1. Other reporter genes originally present in the newly isolated EMS mutants were removed by recombination. **(A)** Control showing normal perlecan distribution in and around the dorsal vessel (dv), in basement membranes of other organs (brain (br), gonad (go), lymph gland (lg), Malpighian tubules (mt)) and at dorsal body wall muscle attachment sites (mas, only partially in focus). **(B)** Homozygous *LanB1*^*S0733*^ with similar perlecan distribution, except for reduced levels and discontinuous lining at the dorsal vessel. **(C-F)** In *Cg25C* mutant embryos perlecan is only partially detectable in a faint BM-like layer in the dorsal vessel and barely detectable in other BMs. Diffuse GFP-perlecan signals are detectable in dorsal and lateral areas of the embryos, some of which overlap with positions of PCs and AMs (arrows). Bright accumulations of GFP-perlecan appear in scattered hemocytes (hc) of homozygous *Cg25C*^*S3064*^**(C)**, *Cg25C*^*DTS-L3*^**(D)** and to a lesser extent in hemizygous *Cg25C*^*DTS-L3*^*/Df(2L)Exel7022***(E)** and type IV collagen null mutants (*Df(2L)Exel7022*, F). **(G, H)** Normal GFP-Vkg distribution in a heterozygous *vkg*^*G454*^*/+* control embryo **(G)** and a *vkg*^*G454*^*/LanB1*^*S0733*^ trans-heterozygote **(H)**. Moderate levels are also observed in hemocytes (hc). **(I)** Trans-heterozygous *vkg*^*G454*^*/Cg25C*^*S3064*^ embryo in which GFP-Vkg is detectable around the dorsal vessel. Presence of Vkg in BMs is partially obscured by high-level accumulation in hemocytes and in the fat body (fb).

The observed embryonic defects of the isolated *LanB1*^*LN*^ mutants are more limited than in laminin null mutants in which BM formation in general is severely affected [45,46]. Hence, the question arises of which matrix structures are still being established in *LanB1*^*LN*^ and *Cg25C* mutants and whether specific defects are seen in or around the dorsal vessel. To answer these questions we investigated the distribution of various ECM components such as laminin, nidogen and perlecan in wild type and mutant embryos. Laminin is produced by various mesodermal and ectodermal cells and in the midgut primordia at extended germ band stage or shortly thereafter, but the bulk of laminin deposition is thought to be derived from expression in migrating hemocytes and the fat body at late stages after germ band retraction (Additional file 7: Figure S3; [35,36,45]). A significant amount of cardiac laminin is probably also derived directly from cardioblasts, which express low levels of *LanB1* mRNA (Additional file 7: Figure S3). Stainings for Laminin protein distribution were performed with a nominal anti-LanB1 antibody, but this antibody is likely to cross-react with another laminin chain as intracellular staining in hemocytes and in the fat body was observed in *LanB1*-deficient embryos (data not shown). In wild-type embryos the lumen and the outer side of the myocardial tube as well as the BMs of other organs such as the gut are contiguously stained by this laminin antibody (Figure 7A,G) or by an antibody against nidogen (Figure 7B,H). In contrast, *LanB1*^*S0733*^ embryos show only residual and frequently spotty laminin and nidogen localization at the luminal and outer side of the cardiac tube (Figure 7C,D). On the other hand, staining around the gut appears more contiguous almost like in the wild type (Figure 7I,J, compare with Figure 7G,H), which is consistent with the absence of severe morphological changes in this tissue. Perlecan incorporation into BMs appears to be affected in a similar manner, as it was only partially detected around the heart tube, while being robustly detected around the gut, CNS, gonad and Malpighian tubules (Figure 8B and data not shown). The prominent presence of BM components around non-cardiac tissues of *LanB1*^*S0733*^ mutants demonstrates that this mutated form of LanB1 is still able to initiate recruitment of other ECM components. However, formation of a contiguous ECM layer is abolished predominantly around the heart tube in *LanB1*^*S0733*^ mutants.

### *Cg25C* mutations impair the incorporation of perlecan into basement membranes

Consistent with the largely normal pericardin distribution and their milder and later occurring defects, *Cg25C* mutants do not share the severe ECM defects observed in *LanB1* mutants. A contiguous ECM layer containing laminin and nidogen forms around the heart tube and around other tissues in homozygous *Cg25C*^*S3064*^ embryos (Figure 7E,F and data not shown). Thus, the glycine exchange G552D does not abolish initial formation of basement membranes, as was observed previously also in *Df(2L)Exel7022* embryos that lack both type IV collagen genes [45]. In spite of this, *Cg25C* mutant embryos feature a very abnormal perlecan distribution. In these mutants, perlecan detectable in form of a GFP-trap fusion protein in live stage 17 embryos (Figure 8C-F) or via immunostaining (data not shown) is incorporated very poorly into the cardiac ECM and basement membranes of other tissues, but is still detected at muscle attachment sites (in outermost Z-sections that are not always included in Figure 8). As shown in Figure 8C-F, GFP-perlecan has a rather diffuse distribution in *Cg25C* mutant embryos (arrows) and is only faintly detectable in the dorsal vessel and around other tissues (compare to Figure 8A). Unlike in the wild type, GFP-perlecan can be found in bright speckles within cells scattered throughout the embryo in mutants with the G552D point mutation, either *Cg25C*^*S3064*^ or *Cg25C*^*DTS-L3*^ (Figure 8C and D, arrowheads) or *Cg25C*^*S3064*^*/Cg25C*^*DTS-L3*^ (not shown). Based on their location and shape these strong signals very likely represent intracellular accumulations of perlecan in hemocytes, which normally express only low levels of perlecan during late embryogenesis [54]. A similar abnormal perlecan distribution was observed in two other glycine mutation alleles (*Cg25C*^*S00120*^ and *Cg25C*^*S0791*^; data not shown), hemizygous mutants (Figure 8E) and in the complete absence of both type IV collagen chains in *Df(2L)Exel7022* embryos (Figure 8F), although perlecan accumulation in hemocytes was less prominent in weak alleles and in the hemizygous and null background.

The aberrant accumulation of perlecan in hemocytes points towards a possible role of collagen not only within the extracellular matrix itself, but also for the secretion of particular components. Notably, observations made with certain semi-dominant mutant forms of collagen Col4a1/a2 from *Caenorhabditis*, mice or human patients with vasculature-related diseases such as hemorrhagic stroke led to the suggestion that particular mutations, most often changing the glycine of a Gly-X-Y repeat, may interfere with the folding and eventually secretion of the heterotrimeric collagen molecule as a whole and may potentially affect also the secretion of other molecules [28,41,55-57]. In order to analyze the distribution of the *Drosophila* Col4a2 homolog Vkg in *Cg25C* missense mutants we made use of the GFP protein trap line *vkg*^*G454*^, which produces a GFP-tagged version of Vkg that is normally incorporated into basement membranes [58,59]. Vkg-GFP was detectable at moderate levels in the ECM of the dorsal vessel (usually with fainter fluorescence signals at the luminal than at the abluminal side), in BMs of other organs and in hemocytes in live stage 17 *vkg*^*G454*^/+ control embryos (Figure 8G) and in heterozygous *LanB1* embryos with one copy of the *LanB1*^*S0733*^ allele (Figure 8H). In heterozygous *Cg25C* embryos that carry one copy the *Cg25C*^*G552D*^ mutation either in the *S3064* (Figure 8I) or *DTS-L3* background (not shown) we observed a striking increase of Vkg-GFP in hemocytes and very strong signals in the fat body. Vkg-GFP was still detectable in basement membranes in most of these embryos, but often only very faintly. Similar intracellular Vkg-GFP enrichments were also observed in other glycine mutation-carrying *Cg25C* alleles, but not upon simple reduction of *Cg25C* gene dosage with a heterozygous collagen IV gene deletion (in *vkg*^*G454*^/*Df(2L)Exel7022* embryos, data not shown), suggesting that a reduced collagen gene dosage alone cannot be made responsible for Vkg-GFP accumulation. Even though *Cg25C/vkg*^*G454*^ genotype combinations do not show morphological defects in embryos, detrimental effects are seen for the alleles with the G552D exchange later on in form of a reduced viability as adults and very poor fertility especially in older females, further supporting the dominant negative nature of Cg25C variants with certain glycine exchanges. Presence of one copy of these *Cg25C* alleles obviously still allows some normally folded collagen IV to be incorporated into BMs at levels sufficient for most developmental processes, but presumably insufficient for full viability, long term muscle integrity and normal oogenesis, a process highly dependent on collagen IV [30,60,61]. As our data show, collagen plays also a particularly critical role in the attachment of pericardial cells and alary muscles to the dorsal vessel and this function goes beyond its own structural contribution to stability as it serves to promote the incorporation of high levels of perlecan.

## Discussion

In a forward genetic screen we recovered several mutants with a primarily cardiac phenotype featuring alary muscle detachments. Mutants belonging to two complementation groups were found to contain domain-specific point mutations in the genes *LanB1* and *Cg25C*, which encode the laminin β chain and collagen IV α1 chain, respectively. Laminin and collagen IV are both essential core constituents of BMs. Their close functional relationship is reflected in the shared primary phenotype of the isolated mutants and in the observed genetic interactions between several mutants of the two complementation groups. Interestingly, several alleles of the collagen IV-encoding genes *Cg25C* and *vkg* obtained in an unrelated EMS screen were later found to interact genetically with each other as well as with certain alleles of the *Laminin A* (*LanA*) gene [62]. However, these mutants have not been molecularly characterized. Although *Drosophila* collagen IV was studied in the context of several developmental questions, no cardiac-related phenotype has been reported for collagen IV α genes in *Drosophila* prior to this work. The role of laminin during embryonic cardiogenesis was investigated to some extent using functional null alleles [6,37,44-46]. Unbiased screening of a large number of EMS mutants allowed us to identify a set of hypomorphic mutations in *LanB1* that are located in the coding region for the LN domain. Mutations within the human *LAMB2* gene are a cause of Pierson syndrome, and while most of those mutations result in premature stop codons and thus can be considered amorphic, the few nonsense mutations found so far also cluster in this domain, underlining its functional importance discussed below [47,63].

### Distinct functions of laminin and collagen in ECM assembly

The severity of cardiac defects, the timing of alary muscle and pericardial cell detachment and the extent to which ECM components are incorporated into the cardiac ECM are consistent with the widely accepted view that laminin is the first ECM component to assemble and that the collagen network will follow thereafter. *Drosophila* embryos devoid of functional laminin trimers due to an amorphic β or γ chain mutation fail to assemble collagen as well as perlecan and nidogen into a dense BM at various tissues [45,46]. Accordingly, mammalian collagen IV was shown to depend on polymerized laminin for its BM incorporation, binding mostly in an indirect way to laminin by using nidogen as a bridge [64-66]. The interrupted arrangement of laminin, nidogen and Prc around the myocardial tube of *LanB1*^*LN*^ embryos implies that cardiac ECM assembly can initiate, but many of those primal areas fail to expand or do not get sufficiently linked in order to persist. In contrast, there is a largely normal layer with basic BM components formed in embryos with the *Cg25C*^*S3064*^ mutation or without any collagen IV (this work and [45]). Regular assembly of laminin and nidogen was previously also detected in larval wing imaginal discs upon RNAi-mediated knockdown of *Cg25C*[67]. On the other hand, the latter approach showed a requirement of collagen IV for perlecan incorporation into the imaginal disc BM, which is akin to our observations in *Cg25C* mutant embryos, in particular regarding the cardiac ECM. In conclusion, collagen IV is not required for the initial steps of cardiac BM assembly in *Drosophila* but for its reinforcement. Similar conclusions have been drawn from studies in other contexts and organisms, e.g. in collagen IV α1/α2-deficient mice [68].

Polymerization among laminin heterotrimers is mediated via the laminin LN domain located on the short arm of each chain (“three-arm model”), but the molecular details of this process are still unclear [66,69,70]. A mutation located in the loop at the tip of the mouse β1 LN domain corresponding to Pierson Syndrome mutation S80R was shown to abolish polymerization in vitro [71]. Since *LanB1*^*LN*^ unlike *LanB1* null mutants display no general loss of BM, but a deteriorating AM/cardiac phenotype during late embryonic stages, we propose that the identified hypomorphic LN mutations, which are conspicuously clustered on one side of the domain (the β6/β3/β8 interface), significantly weaken the intermolecular interaction with at least one of the other short arms, thus preventing the formation of a stable hexagonal laminin network. Alternatively or in addition, binding to other ECM factors could be altered by these mutations.

Supplemented by extensive in vitro studies the sum of the current data suggests a model of stepwise BM assembly [40,66], which in its essence is also applicable to cardiac ECM in *Drosophila*. First, laminin binds to cell surfaces via its α chain C-terminal globular domains that are able to interact with sulfated glycolipids, integrins and dystroglycan. Laminin then polymerizes through its N-terminal LN domains and the formed laminin network serves as a scaffold for direct or nidogen-mediated collagen IV binding. The current model for mammalian BM assembly also includes a feedback towards the cell mediated by binding of the α chain LN domain to integrins or other integral membrane components [40]. BMs are further modified by addition of perlecan and other molecules, some of which might be tissue specific and require additional factors.

### Particular importance of laminin and collagen IV in the ECM of the *Drosophila* dorsal vessel

A weakened ECM around the myocardial tube, although with differences in the molecular details, is ultimately responsible for the closely linked detachment of PCs and AMs in *Cg25C* and *LanB1*^*LN*^ mutants. Because of this prominent cardiac phenotype the isolated mutations should have an impact on certain properties that are more or less specific to the cardiac EMC. One rather simple assumption is that the heart is very sensitive to changes in ECM core components because of its particular structure and function. The dorsal vessel is devoted to pump at high frequency and therefore demands a sturdy, yet highly elastic ECM and a flexible link to associated cells such as PCs and AMs. We have demonstrated that pulling forces from AMs contribute to the detachment and long-range dislocation of PCs from the dorsal vessel, which identifies the ECM between the PCs and CBs as the weakest link. Ultrastructural and immunohistological studies have demonstrated that the dorsal endings of the AMs surround PCs and connect to myocardial cells in an indirect way via an ECM fiber network, which contains Pericardin (Prc) as a specific component [20,23]. Prc has some homology to collagen IV, but its Gly-X-Y repeats in the triple helix-forming domain are more frequently interrupted and preceded by a region with unique atypical repeats [22]. At the current time it is not known whether the different type IV-like collagens, genuine type IV α protomers and the atypical Prc, assemble into mixed multimers. Incorporation of Prc into the cardiac ECM requires the product of the gene *lonely heart* (*loh*), a secreted protein of the ADAMTS-like protein family [23]. However, a failure to interact with Prc alone cannot explain our embryonic AM detachment phenotypes because *prc* mutant embryos still posses attached AMs (data not shown) and cardiac defects of *prc* as well as of *loh* mutants develop later during postembryonic stages [23].

Interestingly, the detachment phenotypes reported herein are very similar to the so-called “broken hearted” (bro) phenotype, which was described in association with mutants of several genes encoding septate junction proteins, factors thought to regulate them, or factors involved in vesicle traffic [72,73]. These phenotypic similarities could perhaps point to a mechanistic connection between factors encoded by the bro genes and core components of the cardiac BM.

Another possible mechanism by which mutations in ubiquitous BM components may lead to specific defects is a disturbed interaction with differentially expressed cell surface receptors. *Drosophila* laminins can bind to integrins, dystroglycan (Dg) and the heparan sulfate proteoglycan syndecan (Sdc), and particular integrins are also known to interact with collagens at least in mammals (summarized in [39,74]). In *Drosophila*, reported genetic interactions between laminin- and integrin-encoding genes, between *vkg* and *αPS3 integrin/scab*, as well as between *Sdc* and *LanA* or *wb* support the notion of functional relationships between BM and integral membrane components [75-77]. Notably, integrins display complex differential expression patterns, while existence of tissue-specific splice variants was documented in the case of Dg [39,78,79]. Integrins are formed by one of five different α subunits (αPS1-5) and one of two β subunits (usually βPS except for the midgut where βν is involved; [80]). LamininA can bind to αPS1/βPS (expressed in the tendon cells, gut epithelium and LVM) and αPS3/βPS (expressed in the dorsal vessel, amnioserosa and midgut), and LamininW can bind to αPS2/βPS (expressed in the trunk visceral mesoderm and body wall muscle attachments) as well as to αPS3/βPS [75,78,81,82]. These differences in integrin binding may lead to distinct phenotypes in *LanB1*^*LN*^ mutants, depending on whether LamininA or LamininW function is more affected and whether redundancy exists in a particular connection.

### Characteristics of *Cg25C* mutations and their implications for development and human diseases

The newly isolated *Cg25C* mutants all contained mutations resulting in glycine changes in one of the Gly-X-Y repeats of the collagen triple helix-forming domain. The identification of functionally important glycine residues within the collagen IV α1 chain adds to the growing evidence that certain collagen mutations may exert a semi-dominant effect leading to an overall weakening of ECM structures and intracellular accumulation of the collagen protomer and other molecules, both of which could contribute to collagen-related diseases including vascular and cardiac abnormalities [41,43].

According to in vitro studies and observations originally made on the fibrillar type I collagen, glycine mutations in the Gly-X-Y repeats are expected to inhibit folding and stability of the triple-helical structure, promote excessive modifications and hinder collagen protomer secretion [83,84]. The *Cg25C* mutants differ in the severity of their phenotype and their ability to reduce viability in trans with the *LanB1* alleles. The strongest *Cg25C* alleles, *Cg25C*^*S3064*^ and the recently described *Cg25C*^*DTS-L3*^ mutant, which carry the same G552D mutation, showed genetic interaction with *vkg*^*01209*^ and all our *LanB1* EMS alleles, but not with any of the eight *laminin α1,2*/*wb* alleles that were also isolated in the screen. The identity of the mutations in these two independent lines appears highly coincidental but may highlight a particularly critical residue, especially as this mutation shows characteristics of a temperature-sensitive antimorph [30]. Non-random occurrence of glycine mutations after EMS mutagenesis screens were also noticed in the α1 (*emb-9*) and α2 (*let-2*) collagen IV genes of *Caenorhabditis elegans*[28,85-87]. Like in our case, several of these mutations were described as semi-dominant temperature-sensitive. A visible defect of these mutants was detachment of muscle fibers from the hypodermis at about the same stage as muscles start to contract. Similar body wall muscle detachments have been observed in *Drosophila* after forced expression of a dominant-negative *Cg25C* transgene with an internal deletion in the triple helical region [88]. In contrast to this artificially generated version, our *Cg25C* EMS alleles and the *Cg25C*- and *vkg*-deleting deficiency *Exel7022* do not show significant defects in the body wall musculature until final stages of embryonic development. At later stages, heterozygous *Cg25C*^*DTS-L3 (G552D)*^*/+* animals show aberrations in the sarcomeric ultrastructure of larval body wall muscles and in the adult oviduct musculature if grown at 29°C, thus arguing for a function of *Cg25C* in maintaining the integrity of several muscle types [30].

Dominant phenotypes that are stronger in point mutants than in null mutants suggest that the aberrant collagen chain interferes with the function of other molecules, e.g. by reducing the export of Vkg. This would explain the changes in Vkg-GFP distribution in heterozygous *Cg25C*^*S3064*^*/+* and *Cg25C*^*S0120*^*/+* embryos, which feature weaker signals in BMs and strongly increased Vkg-GFP within cells of the fat body and in hemocytes. In another study, RNAi-mediated knockdown of *Cg25C* in the larval fat body caused diffuse accumulation of Vkg-GFP in the hemolymph and prevented its deposition into BMs, indicating that, in the absence of Cg25C, Vkg is secreted to the hemolymph in nonfunctional monomeric form [67]. Intracellular accumulation of type IV collagen and concomitant decrease of BM-localized collagen was detected in *Caenorhabditis* mutants carrying glycine substitutions in their collagen chains, in particular at elevated temperature [28]. Similar negative effects on collagen IV secretion have also been demonstrated in homozygous or heterozygous *Col1a* mutant mice with a short internal deletion in the triple helix-forming domain, which additionally was accompanied by an increase in several endoplasmic reticulum (ER)-resident proteins [55,89]. This suggests that semi-dominant forms of collagen could also affect the secretion of other molecules, which would be consistent with our detection of high levels of perlecan-GFP in hemocytes of strong *Cg25C* mutants. This does not exclude the suggested extracellular role of collagen IV for perlecan BM localization, since diffuse distribution of perlecan-GFP throughout the embryo and hardly any BM incorporation were observed in collagen IV-deficient embryos. Considering the dramatic increase of Vkg-GFP in embryos heterozygous for strong glycine mutation alleles of *Cg25C*, we cannot rule out that this accumulation is connected to a net increase in collagen chain synthesis due some a feedback mechanism induced by misfolded matrix components or monomeric Vkg either directly within the ER or via an unknown signaling mechanism from the extracellular space.

Type IV collagen mutations, most of them causing glycine changes in Gly-X-Y motifs, were also found in human *COL4A1* and with lesser frequency in *COL4A2* in connection with various diseases such as porencephaly, hemorrhagic stroke, small vessel disease and the Hereditary Angiopathy with Nephropathy, Aneurysms, and Muscle Cramps (HANAC) Syndrome [41,43,90]. The molecular mechanism underlying these diseases is still a matter of debate and may depend on the particular allele. In some cases intracellular accumulation of collagen IV and induction of ER stress were demonstrated, while other data point towards specific dominant-negative interactions within the ECM [41,91]. Further work is required in order to fully understand the mechanisms by which collagen mutations interfere with normal development and tissue integrity.

## Conclusions

The isolation of randomly induced point mutations in genes encoding core basement membrane components demonstrates that the dorsal vessel and its linkage to pericardial cells and alary muscle fibers are highly dependent on a perfectly assembled ECM. Pulling forces from alary muscles contribute to the detachment and long-range dislocation of pericardial cells from the dorsal vessel in which the ECM has been weakened by mutations affecting ECM components. Mutations can weaken the ECM around the myocardial tube by different mechanisms. Our phenotypic analyses support current models of stepwise basement membrane assembly, in which laminin plays a primary role, whereas collagen IV is needed for the stabilization of ECM structures. Accordingly, amorphic mutations in genes encoding unique laminin chains (*LanB1* and *LanB2*), which do not permit the production of functional laminin heterotrimers, will abolish initiation of basement membrane formation and therefore will cause more pleiotropic phenotypes. Isolation of a set of clustered hypomorphic mutations in *LanB1* suggests that the laminin β LN domain is particularly crucial for formation of a contiguous ECM around and within the heart and therefore for myocardial lumen formation, myocardial tube stability and attachment of pericardial cells and alary muscles. We suggest that in these *LanB1*^*LN*^ mutants laminin polymerization is impaired due to the amino acid substitutions at the β6/β3/β8 interface of the LN domain. The LanA (α3,5) short arm is a likely binding partner of this interface, since loss of *LanA* causes a phenotype similar to that of *LanB1*^*LN*^ mutants (mostly affecting the heart tube and alary muscle attachment, but not the gut). Wb/α1,2-containing LamininW molecules may partially allow ECM assembly in some non-cardiac tissues, possibly due to different requirements for laminin network formation and differential receptor binding.

Our EMS screen revealed that *Cg25C* encoding the type IV collagen α1 chain is vital for a stable attachment of pericardial cells and alary muscles to the dorsal vessel. Collagen IV ensures normal incorporation of perlecan into embryonic basement membranes and this function also applies to the more elaborated ECM of the dorsal vessel. Mutations causing exchanges of glycine in certain Gly-X-Y repeats of the collagen IV triple helix-forming domain may exert a semi-dominant effect leading to less stable ECM structures as well as intracellular accumulation of collagen and possibly other molecules, thus paralleling observations made in other organisms and in connection with collagen-related diseases. The detected temperature-sensitivity of the phenotypes in our *Cg25C* EMS mutants fits well with observations in other organisms, reinforcing the idea that this is a rather widespread phenomenon of collagen glycine mutations.

## Methods

### Isolation of EMS mutants

In order to detect muscle defects in an EMS mutagenesis screen we constructed a RFP/GFP reporter line, which expresses RFP driven by the *HN18* somatic muscle enhancer of *org-1* (H. Nagaso, unpublished; contains the enhancer *HN39* described in [18]) and by the longitudinal visceral muscle (LVM)-specific *HLH54Fb* enhancer of *HLH54F*[92], as well as GFP driven by two copies of a cardioblast-specific enhancer of *tinman* (tinC*, which in contrast to endogenous *tin* is active in all cardioblasts) [93,94]. Individual transgenes selected for high expression, viability and normal development were combined via recombination in a *y*^*−*^*w*^*−*^ background using the mini-*w*^*+*^ marker of the P{RedH-Pelican}-based RFP reporter constructs and the *y*^*+*^ marker contained in the tinC*-GFP construct pGD130 (gift from G. Dietzl and F. Schnorrer). For mutagenesis the targeted chromosome 2 carried the following three reporter insertions: *P{RedH-Pelican.org-1-HN18-dsRed}18a* (in *Jon25Bi*, one of three paralogs in region 25B), P{pGD130.tinC*-GFP}13b (in *cn*) and *P{RedH-Pelican.HLH54Fb-dsRed}16c* (630 bp upstream of *qsm-RA*) (or for lines *S0001-S0800*: *P{RedH-Pelican.HLH54Fb-dsRed}16b*, inserted in the first large intron of *Sin3A*). For brevity, the reference strains were named according to the second chromosome insertion sites and recombinant number *S-18a-13b-16c.1* and *S-18a-13b-16b.1*, respectively. Homozygous flies were mutagenized with 25-35 mM EMS according to standard procedures resulting in an average frequency of 2.1 lethal hits per chromosome (assuming Poisson distribution) and screening was performed as described previously [95-97]. In total over 3700 lines were analyzed for embryonic defects in cardiac, somatic or visceral musculature. Mutant lines were maintained over a “green balancer” (*CyO, P{w[+mC] = GAL4-twi.G}2.2, P{UAS-2xEGFP}AH2.2*) to allow recognition of GFP-negative homozygotes.

### *Drosophila* stocks

The following mutant *Drosophila melanogaster* strains were used for mapping and characterization of the reported EMS alleles: *Cg25C*^*DTS-L3*^ and *Cg25C*^*b-9*^ ([30], gift from M. Mink, University of Szeged), *LanA*^*9–32*^ ([98], gift from T. Volk, Weizmann Institute of Science), *P{lacW}Cg25C*^*k00405*^, *P{SUPor-P}LanB1*^*KG03456*^, *Mi{MIC}LanA*^*MI02491*^*, Mi{ET1}LanA*^*MB01129*^*, Mi{MIC}LanB2*^*MI03747*^*, Mi{ET1}prc*^*MB03017*^, *P{PZ}vkg*^*01209*^, *P{PZ}wb*^*09437*^, *Df(2L)BSC110*, *Df(2L)BSC172*, *Df(2L)Exel7022*, *Df(2L)BSC233*, *Df(2L)ED12527* and about 180 additional deficiencies spanning chromosome 2 (all available from the Bloomington Stock Center at Indiana University, USA). For phenotypic analysis we used these additional reporter lines: *Hand-GFP* on chromosome 3 (*HCH-GFP*; [51]), *Mhc-tau::GFP* on chromosome X ([99]; obtained from F. Schnorrer, Max-Planck-Institute of Biochemistry) and the Flytrap GFP lines *vkg*^*G454*^ (*vkg::GFP*) and *trol*^*ZCL1973*^ (*trol::GFP*) ([58,59,100], obtained from L. Cooley, Yale University Medical School). For the analysis of pericardial cells in the absence of alary muscles, mutants of the desired allele were combined with the X-chromosomal mutation *org-1*^*OJ487*^[18] and crossed with males of the corresponding single mutant allele carrying *Hand-GFP*.

### Mapping of *LanB1* and *Cg25C* alleles

Novel *LanB1* and *Cg25C* alleles were initially identified by complementation tests (routinely performed at 25°C unless stated otherwise) using either a candidate gene approach, which resulted in the identification of the strong *LanB1* and *wb* alleles, or by unbiased complementation crosses with deficiencies covering most of chromosome 2. The unbiased method was performed for lines *S0733*, *S1348* and *S3064*. Deficiencies lethal or semi-lethal with the investigated lines were tested for the occurrence of visible embryonic phenotypes using the GFP/RFP reporters present on the mutated chromosome. Furthermore, complementation of lethality and the embryonic phenotype was analyzed in trans with other available deficiencies and mutants of the confined region. Similar looking alleles of our screen were crossed with each other and later with identified deficiencies or mutants to establish complementation groups.

The presence of *LanB1* and *Cg25C* mutations was eventually confirmed by sequencing of the *LanB1* and *Cg25C* locus, respectively. Sequencing was done from PCR products amplified from genomic DNA isolated from homozygous *twi>>*GFP-negative stage 12–16 embryos. All mutations were verified by sequencing of at least 2 independent DNA preparations. DNA from the unmutagenized RFP/GFP reporter strains *S-18a-13b-16c.1* and *S-18a-13b-16b.1* served as references. For *Cg25C*, one additional sequence difference (Pro 1523 to Ala) in comparison to the annotated sequence AAN10520.1 was present in all investigated lines as well as in the unmutagenized chromosome, and therefore is considered a polymorphism.

### Embryo mountings for GFP/RFP live fluorescent analysis

Standard egg collections were performed at 25°C unless noted otherwise. Eggs were dechorionated with bleach for 2 minutes and rinsed with water in collection baskets or in custom made 3x5 arrays for high-throughput. For screening and acquisition of GFP/RFP still images embryos were mounted in 16% glycerol using a brush. For time-lapse imaging embryos carrying GFP/RFP reporter genes were dechorionated, aligned on an agar block, transferred to a cover slip with a line of glue and covered with a small drop of halocarbone oil and an air-permeable membrane (High Sense, Oxygen Probe Service Kit, YSI Incorporated, Ohio, USA). Time-lapse image series were acquired on a Leica SP5 II confocal system using a HC PL APO20x/0.70 objective (with glycerol), an argon laser for excitation at 488 nm and detection at 500–550 nm and a DPSS 561 laser for excitation at 561 nm and detection at 570–700 nm. Acquisition was done over a time course of about 6–10 hours with the following settings: 1.8x optical zoom, scan speed 200 Hz, line averaging 3, resolution 1024 × 600 pixel, Z-stack of about 20 sections with a step size of 3 μm, and time intervals of about 2 minutes per stack. Movies were generated using Leica Application Suite Advanced Fluorescence (LAS-AF) 2.4.1 and ImageJ 1.46 software.

### Staining procedures

Embryos were fixed and immunostained for proteins using fluorescently labeled secondary antibodies or if necessary with enhancement by VectaStain Elite ABC kit (Vector Laboratories) and tyramide (TSA, PerkinElmer Inc., as indicated) using standard procedures as previously described [16,101]. Alternative heat fixation was performed prior to staining with boiling buffer (68 mM NaCl, 0.04% Triton X-100) and heptane/methanol (1:1) after the protocol described in [102]. The following antibodies were used: rabbit anti-Dg^Cterm^ (1:1200) [79], rabbit anti-GFP (1:2000) (Invitrogen, A6455), mouse anti-GFP (1:200, TSA) (Invitrogen, A11120), rabbit anti-LanB1 (1:1000) (Abcam, ab47650), rabbit anti-Mef2 (1:1500) (from H.T. Nguyen, Erlangen University), rabbit anti-Ndg (1:1000) [103], rat anti-Odd (1:600, TSA) [104], rabbit anti-Perlecan (1:1000) [105], mouse anti-Prc EC11 (1:10, TSA) (Developmental Studies Hybridoma Bank, DSHB, University of Iowa), rabbit anti-RFP (1:300) (Abcam), mouse anti-Slit C555.6D (1:10, TSA) (DSHB) and rabbit anti-Tin (1:750) [93]. RNA in situ hybridizations were carried out in combination with protein immunostainings essentially as previously described [101]. PCR-amplified DNA-Fragments of the genes *Cg25C* (1401 nt; primers Cg25C-F08 TCGGCGGCAAATGCTCATCG and T7-Cg25C-B09 TAATACGACTCACTATAGGTTTCCAGGAGCACCACGGTCAC) and LanB1 (2400 nt; primers LanB1-F06 GGACAACTTCTTTGGCAATCCG and T7-LanB1-B07 TAATACGACTCACTATAGGGGTGGCACTTGTGGCAGACTG) were used to generate digoxigenine-labeled antisense RNA probes. Images were acquired using a Zeiss Apotome or a Leica SP5 II laser scanning confocal microscope system.

### Availability of supporting data

The data set supporting the results of this article is included within the article and its additional files.

## Abbreviations

ECM: Extracellular matrix; BM: Basement membrane; CM: Cardiomyocyte; PC: Pericardial cell; AM: Alary muscle; LVM: Longitudinal visceral musculature.

## Competing interests

The authors declare that they have no competing interests.

## Authors’ contributions

DH designed and performed experiments and analyzed the data. MF designed the EMS screen, provided input in data interpretation and helped in drafting the manuscript. IR designed the EMS screen and conceived subsequent experiments, performed experiments, analyzed the data and drafted the manuscript. All authors read and approved the final manuscript.

## Supplementary Material

Additional file 1: Figure S1 Comparison of the phenotypes of embryos with mutations in the different laminin chain genes. Live preparations of stage 17 embryos mutant for the different laminin chain genes with GFP and RFP markers as in Figure1A. (A) Wild type control embryo. (B) Homozygous *LanB1*^*S1163*^ embryo with beginning AM detachment (arrows). The dorsal vessel displays an abnormal morphology (arrowhead), but has not yet retracted. (C) Homozygous *LanB1*^*S3773*^ embryos show similar defects, but frequently retain AM/DV attachment at the posterior end even at very late stages. In the two hypomorphic alleles, *LanB1*^*S1163*^ (B) and *LanB1*^*S3773*^ (C), the midgut is mostly constricted and looped and entirely surrounded by LVM fibers, essentially as in the control. (D, E) The amorphic alleles *LanB1*^*S0473*^ and *LanB1*^*S1522*^ show a more pleiotropic phenotype that includes severe heart defects (arrowheads) and lack of midgut constrictions. Large portions of the midgut are not associated with visceral musculature (*). (F) Embryo with a gene-disrupting insertion in the laminin γ-chain coding gene *LanB2*. The phenotype is virtually indistinguishable from that of amorphic *LanB1* alleles. (G, H) The EMS-induced *wb* (*laminin α1,2*) mutants *wb*^*S0374*^ and *wb*^*S1425*^ show prominent midgut/LVM defects and in some cases myocardial gaps (arrowheads), but opposing cardiomyocytes are mostly separated by a luminal space (thin arrow). LMV fibers are missing in some areas of the partially unconstricted midgut (*) or are bunched together (brackets). In most cases AM attachment is normal. (I, J) In contrast to *wb* mutants, homozygous *LanA* (*laminin α3,5*) mutants feature detachment of alary muscles (arrows) and strong heart tube defects (arrowheads) similar to *LanB1*^*LN*^ mutants. Gut morphology and LVM arrangement are mostly unaffected. Click here for file

Additional file 2: Figure S2 Temperature sensitivity of the alary muscle detachment phenotype in *Cg25C* alleles. Live preparations of *Cg25C* mutant embryos with GFP and RFP markers as in Figure 1A and Figure 3A raised at the indicated temperature. (A, B) Trans-allelic *Cg25C*^*S3064*^*/Cg25C*^*S0791*^ embryos show normal alary muscle (AM) attachment at 19°C, but AM detachment at 29°C. (C, D) Hemizygous *Cg25C*^*S2186*^*/Df(2L)Exel7022* embryos show normal AM attachment at 25°C, but AM detachment at 29°C. Click here for file

Additional file 3: Movie S1 Time-lapse recording of normal migration of heart cells and alary muscles during dorsal closure. Migration of alary muscles (AMs) and heart cells in a wild type embryo imaged during stages 15 to 17 (dorsal view). AMs are visualized by *org-1*-*SM*-RFP (red); cardioblasts, pericardial cells and anteriorly (to the left) the lymph gland are labeled by *Hand*-GFP (green). Additional *Hand*-GFP-positive nuclei of the circular visceral musculature are close to the fibers of the longitudinal visceral musculature marked by *HLH54F-LVM*-RFP (red). Note the continuous close contact between migrating cardioblasts and pericardial cells. Click here for file

Additional file 4: Movie S2 Heart cell and alary muscle migration in *LanB1*^*S0733*^ mutants. Time-lapse recording of a homozygous *LanB1*^*S0733*^ embryo during stages 15 to 17 with slight irregularities in the migration of *Hand*-GFP-labeled PCs prior to heart tube closure (arrowheads). Shortly before tube formation the posterior pair of the *org-1-SM*-RFP-labeled AMs loses contact with the heart. The detaching AMs pull several PCs towards lateral positions at the end of embryonic development (arrows). Click here for file

Additional file 5: Movie S3 Heart cell and alary muscle migration in another *LanB1*^*S0733*^ embryo focusing on the posterior part of the developing heart. After tube closure the posterior AMs start to detach from the heart. The dorsal vessel loses its posterior attachment and the distance to the AMs grows. The detaching AMs pull away small groups of PCs similar as in Movie S2 (arrows). Click here for file

Additional file 6: Movie S4 Heart cell migration in the absence of alary muscles. In *org-1*^*OJ487*^; *LanB1*^*S0733*^ double mutant embryos heart cells (labeled by *Hand*-GFP) are correctly specified but AM fibers are missing. At stages 15 to 17 some dissociated PCs are observed at intermediate distance to the cardiomyocytes (arrowheads), only a single PC can be found at large distance to the heart (arrow). Click here for file

Additional file 7: Figure S3 Embryonic expression of *LanB1* and *Cg25C*. Expression of *LanB1* (A-C) and *Cg25C* (D-F) RNA (green, as indicated) detected by in situ hybridization in wild type embryos (A, B, D, E) and *LanB1* (C) or *Cg25C* (F) deficient embryos. All embryos were additionally stained for Mef2 (red) to identify rows of cardioblasts/cardiomyocytes (ca). (A) Lateral view of a stage 14 embryo with clearly identifiable *LanB1* expression in hemocytes (hc) and the amnioserosa (as). (B) Dorsal view of a stage 16 embryo with strong *LanB1* expression in the fat body (fb) and hemocytes. (A’, B’) Higher magnifications of the cardiac area demonstrate presence of *LanB1* RNA within and adjacent to cardiac cells. (C, C’) Dorsal view of a homozygous *Df(2L)ED12527* embryo at stage 16. In this *LanB1* null mutant no *LanB1* RNA is produced and only artificial dots at segment borders are visible. (D, D’) At stage 14 *Cg25C* RNA is strongly expressed in hemocytes and weakly in cardioblasts. (E, E’) Dorsal view of a stage 16 embryo with strong *Cg25C* expression in hemocytes and the fat body and moderate expression in cardiomyocytes. (F, F’) Dorsal view of a stage 16 *Cg25C*-deficient *Df(2L)Exel7022* embryo without any *Cg25C* expression. Click here for file
