# Supplementary figures and images for: Distinct functions of the laminin β LN domain and collagen IV during cardiac extracellular matrix formation and stabilization of alary muscle attachments revealed by EMS mutagenesis in Drosophila
Source: BMC Dev Biol. 2014 Jun 17;14:26. doi: 10.1186/1471-213X-14-26 (PMC4068974; doi:10.1186/1471-213X-14-26)

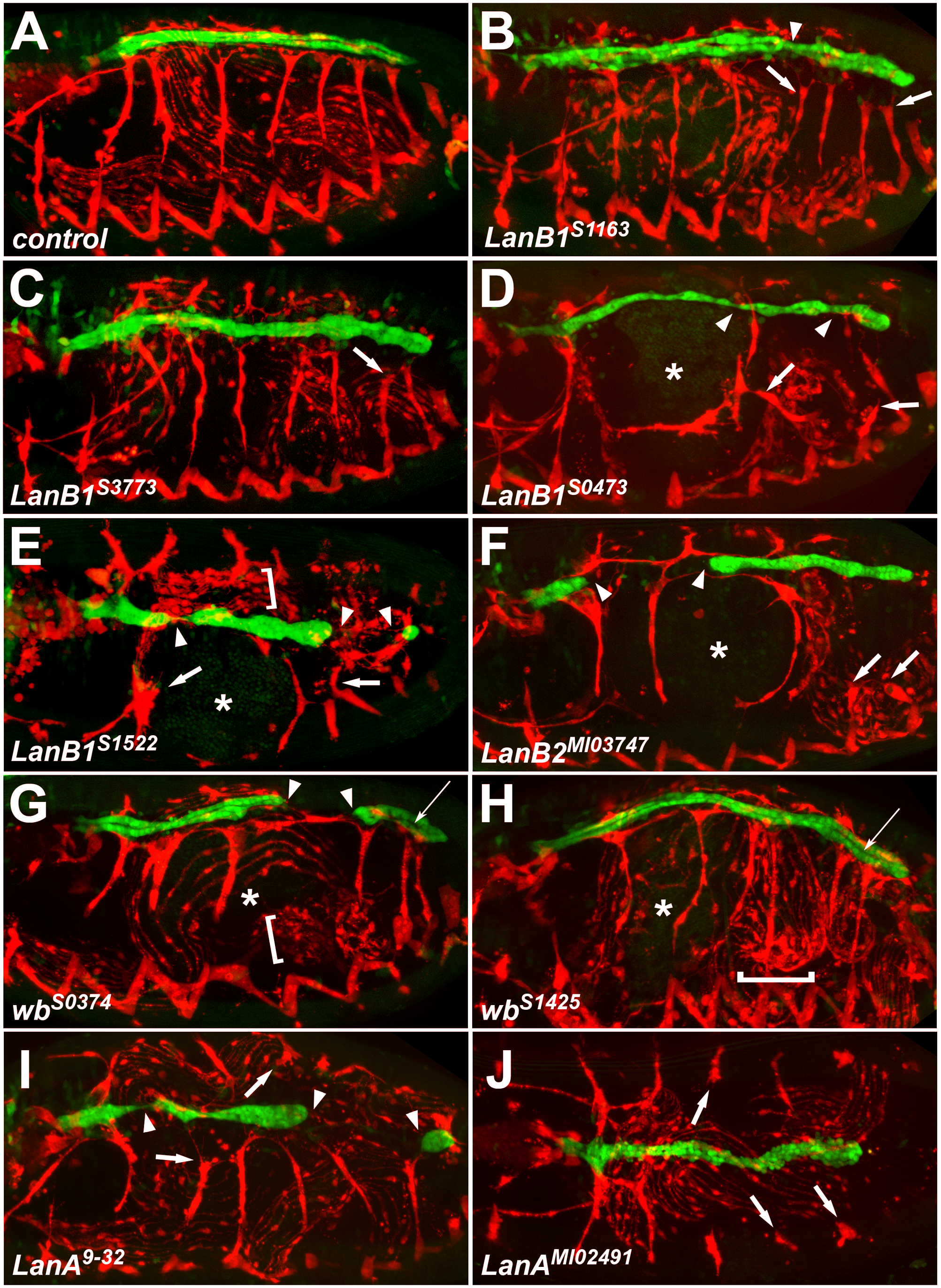

Supplement: Additional file 1: Figure S1 — Comparison of the phenotypes of embryos with mutations in the different laminin chain genes. Live preparations of stage 17 embryos mutant for the different laminin chain genes with GFP and RFP markers as in Figure1A. (A) Wild type control embryo. (B) Homozygous LanB1 S1163 embryo with beginning AM detachment (arrows). The dorsal vessel displays an abnormal morphology (arrowhead), but has not yet retracted. (C) Homozygous LanB1 S3773 embryos show similar defects, but frequently retain AM/DV attachment at the posterior end even at very late stages. In the two hypomorphic alleles, LanB1 S1163 (B) and LanB1 S3773 (C), the midgut is mostly constricted and looped and entirely surrounded by LVM fibers, essentially as in the control. (D, E) The amorphic alleles LanB1 S0473 and LanB1 S1522 show a more pleiotropic phenotype that includes severe heart defects (arrowheads) and lack of midgut constrictions. Large portions of the midgut are not associated with visceral musculature (*). (F) Embryo with a gene-disrupting insertion in the laminin γ-chain coding gene LanB2. The phenotype is virtually indistinguishable from that of amorphic LanB1 alleles. (G, H) The EMS-induced wb (laminin α1,2) mutants wb S0374 and wb S1425 show prominent midgut/LVM defects and in some cases myocardial gaps (arrowheads), but opposing cardiomyocytes are mostly separated by a luminal space (thin arrow). LMV fibers are missing in some areas of the partially unconstricted midgut (*) or are bunched together (brackets). In most cases AM attachment is normal. (I, J) In contrast to wb mutants, homozygous LanA (laminin α3,5) mutants feature detachment of alary muscles (arrows) and strong heart tube defects (arrowheads) similar to LanB1 LN mutants. Gut morphology and LVM arrangement are mostly unaffected. [file 1471-213X-14-26-S1.tiff]

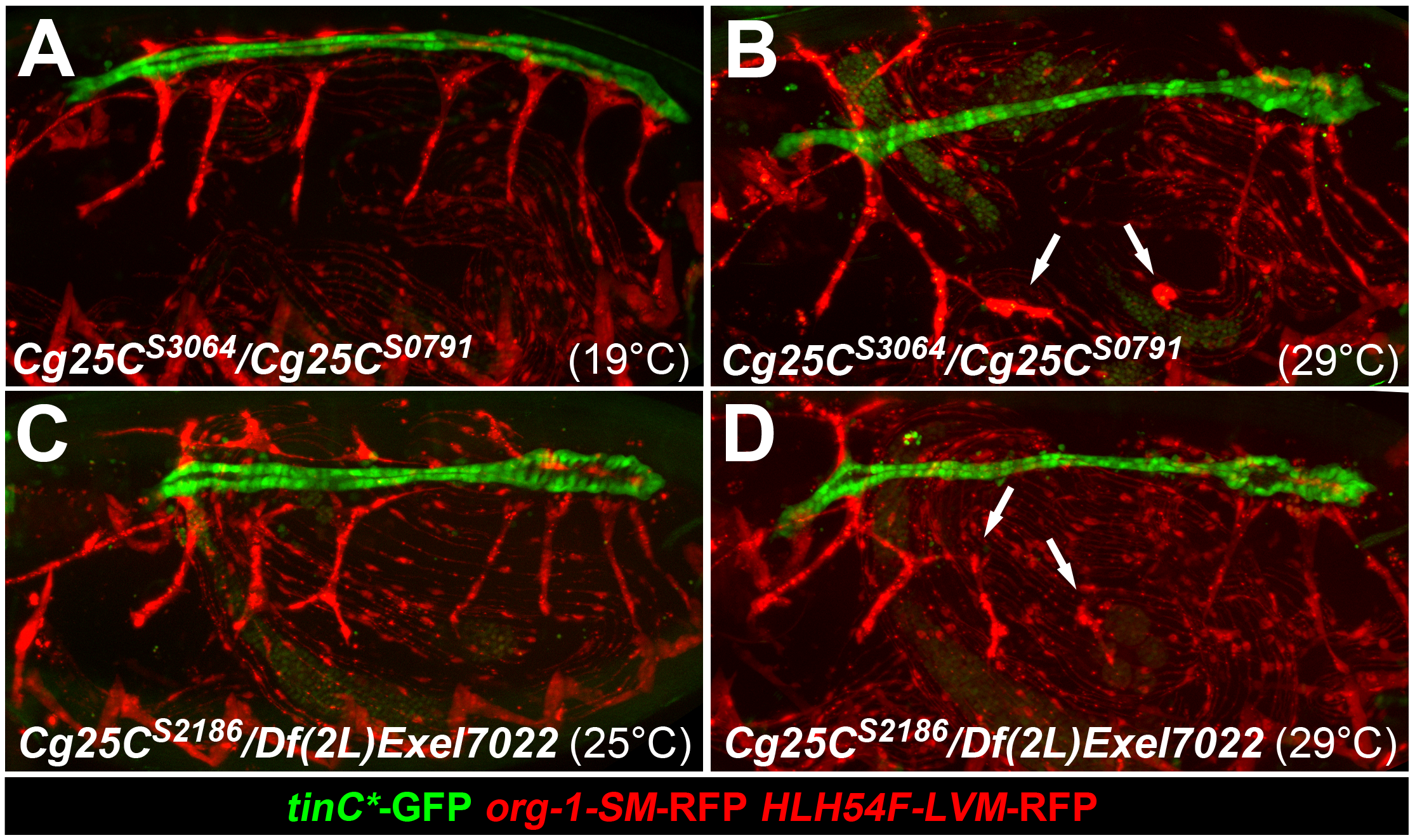

Supplement: Additional file 2: Figure S2 — Temperature sensitivity of the alary muscle detachment phenotype in Cg25C alleles. Live preparations of Cg25C mutant embryos with GFP and RFP markers as in Figure 1A and Figure 3A raised at the indicated temperature. (A, B) Trans-allelic Cg25C S3064 /Cg25C S0791 embryos show normal alary muscle (AM) attachment at 19°C, but AM detachment at 29°C. (C, D) Hemizygous Cg25C S2186 /Df(2L)Exel7022 embryos show normal AM attachment at 25°C, but AM detachment at 29°C. [file 1471-213X-14-26-S2.tiff]

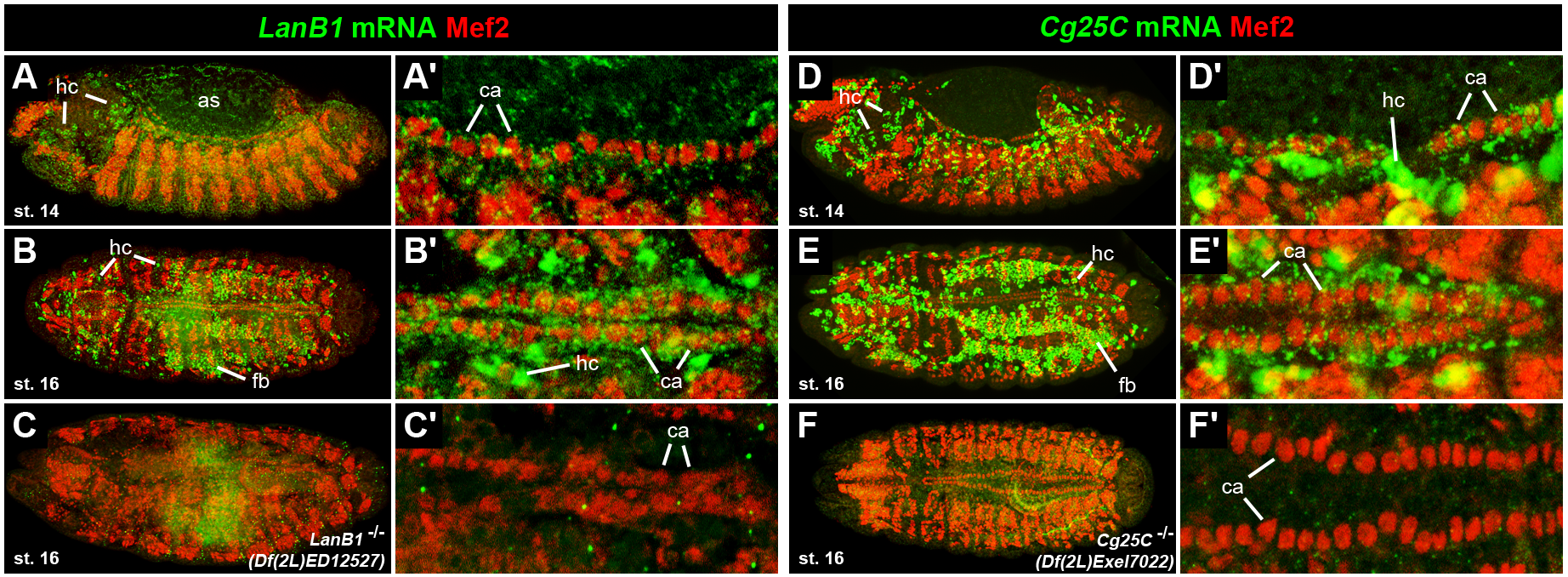

Supplement: Additional file 7: Figure S3 — Embryonic expression of LanB1 and Cg25C. Expression of LanB1 (A-C) and Cg25C (D-F) RNA (green, as indicated) detected by in situ hybridization in wild type embryos (A, B, D, E) and LanB1 (C) or Cg25C (F) deficient embryos. All embryos were additionally stained for Mef2 (red) to identify rows of cardioblasts/cardiomyocytes (ca). (A) Lateral view of a stage 14 embryo with clearly identifiable LanB1 expression in hemocytes (hc) and the amnioserosa (as). (B) Dorsal view of a stage 16 embryo with strong LanB1 expression in the fat body (fb) and hemocytes. (A’, B’) Higher magnifications of the cardiac area demonstrate presence of LanB1 RNA within and adjacent to cardiac cells. (C, C’) Dorsal view of a homozygous Df(2L)ED12527 embryo at stage 16. In this LanB1 null mutant no LanB1 RNA is produced and only artificial dots at segment borders are visible. (D, D’) At stage 14 Cg25C RNA is strongly expressed in hemocytes and weakly in cardioblasts. (E, E’) Dorsal view of a stage 16 embryo with strong Cg25C expression in hemocytes and the fat body and moderate expression in cardiomyocytes. (F, F’) Dorsal view of a stage 16 Cg25C-deficient Df(2L)Exel7022 embryo without any Cg25C expression. [file 1471-213X-14-26-S7.tiff]
